# Cyclic AMP signalling controls key components of malaria parasite host cell invasion machinery

**DOI:** 10.1371/journal.pbio.3000264

**Published:** 2019-05-10

**Authors:** Avnish Patel, Abigail J. Perrin, Helen R. Flynn, Claudine Bisson, Chrislaine Withers-Martinez, Moritz Treeck, Christian Flueck, Giuseppe Nicastro, Stephen R. Martin, Andres Ramos, Tim W. Gilberger, Ambrosius P. Snijders, Michael J. Blackman, David A. Baker

**Affiliations:** 1 Faculty of Infectious Diseases, London School of Hygiene & Tropical Medicine, London, United Kingdom; 2 Malaria Biochemistry Laboratory, The Francis Crick Institute, London, United Kingdom; 3 Mass Spectrometry Proteomics Platform, The Francis Crick Institute, London, United Kingdom; 4 Crystallography, Institute of Structural and Molecular Biology, Birkbeck College, London, United Kingdom; 5 Signalling in Apicomplexan Parasites Laboratory, The Francis Crick Institute, London, United Kingdom; 6 Macromolecular Structure Laboratory, The Francis Crick Institute, London, United Kingdom; 7 Institute of Structural and Molecular Biology, University College London, London, United Kingdom; 8 Bernhard-Nocht Institute for Tropical Medicine, Hamburg, Germany; Albert Einstein College of Medicine, UNITED STATES

## Abstract

Cyclic AMP (cAMP) is an important signalling molecule across evolution, but its role in malaria parasites is poorly understood. We have investigated the role of cAMP in asexual blood stage development of *Plasmodium falciparum* through conditional disruption of adenylyl cyclase beta (ACβ) and its downstream effector, cAMP-dependent protein kinase (PKA). We show that both production of cAMP and activity of PKA are critical for erythrocyte invasion, whilst key developmental steps that precede invasion still take place in the absence of cAMP-dependent signalling. We also show that another parasite protein with putative cyclic nucleotide binding sites, *Plasmodium falciparum* EPAC (PfEpac), does not play an essential role in blood stages. We identify and quantify numerous sites, phosphorylation of which is dependent on cAMP signalling, and we provide mechanistic insight as to how cAMP-dependent phosphorylation of the cytoplasmic domain of the essential invasion adhesin apical membrane antigen 1 (AMA1) regulates erythrocyte invasion.

## Abstract 2

Conditional gene knockout shows that cyclic AMP signalling is absolutely required for invasion of erythrocytes by malaria parasites. Quantitative mass spectrometry identifies the parasite proteins whose phosphorylation is adversely affected when cyclic AMP signalling is ablated.

## Introduction

Malaria kills over 400,000 people each year across the world. Despite significant reductions in deaths and clinical cases of malaria between 2000 and 2015 [1], these numbers have now plateaued, and efforts to eliminate the disease are threatened by the emergence of drug-resistant *Plasmodium* strains. New interventions are urgently needed to strengthen malaria control and to prevent global malaria incidence and mortality rates from rising again. Malaria pathology is caused by the asexual blood stages of the parasite life cycle. In *P*. *falciparum*, the most lethal species of malaria parasite, blood stage development is characterised by a 48-h cycle. This begins with a rapid invasion step, in which merozoites enter erythrocytes and convert into ring-stage forms within a membrane-bound parasitophorous vacuole (PV). The ring forms transform into trophozoites, which digest haemoglobin and begin replicating their DNA. The resulting schizonts undergo segmentation to produce merozoites that burst out of the red cell in a highly regulated process called egress. Within seconds to minutes of egress, merozoites invade new host erythrocytes. An understanding of the molecular bases of the critical developmental steps involved in egress and invasion is required to advise the rational design of much-needed novel therapeutics targeting the malaria parasite.

Egress is triggered by elevated cyclic GMP (cGMP) levels that activate the single parasite cGMP-dependent protein kinase (PKG) [2]. Over 100 sites in approximately 70 *P*. *falciparum* schizont proteins are thought to be phosphorylated following PKG activation [3], but it is not known which of these phosphorylation events are key to merozoite egress and subsequent steps in the life cycle. PKG activity is required for the discharge of organelles known as exonemes [2], releasing a proteolytic enzyme called subtilisin-like protease 1 (SUB1), which cleaves a number of proteins that have major downstream roles in merozoite egress and invasion [4–7]. PKG activity is also required for mobilisation of calcium from intracellular stores [8] and the subsequent activation of calcium-dependent protein kinases (CDPKs). These in turn are thought to be required for the discharge of a second set of apical organelles called micronemes [9–11], which contain proteins with key roles in invasion.

Alongside these known roles of PKG in egress and ‘priming’ of merozoites for invasion, the single parasite cyclic AMP (cAMP)-dependent protein kinase (PKA), composed of catalytic and regulatory subunits respectively called PKAc and PKAr, is also thought to play a role in invasion. Bioinformatic analyses of *P*. *falciparum* schizont and merozoite phosphoproteome data have suggested the involvement of PKA-mediated signalling in a range of cellular processes, including activation of a merozoite actinomyosin-based molecular motor required for invasion [12,13]. Adenylyl cyclase beta (ACβ), an orthologue of the mammalian soluble adenylyl cyclase, is thought to be the only enzyme by which asexual blood stage malaria parasites synthesise cAMP and thereby activate PKA [14]; adenylyl cyclase alpha (ACα) is not expressed in blood stage malaria parasites but is thought to have a role in liver cell invasion by sporozoites [15]. Pharmacological inhibition of ACβ has been reported to prevent the release of calcium from intracellular stores, thus inhibiting microneme secretion and invasion [16]. However, the findings of that study suggested that the observed cAMP-dependent increase in cytosolic calcium was independent of PKA activity, instead operating through an exchange protein directly activated by cAMP (EPAC), a molecule that in mammalian cells binds to cAMP and triggers calcium release through interaction with the small G protein Ras-related protein 1 (RAP1) and activation of a phosphatidylinositol-specific phospholipase C (PI-PLC)-dependent pathway. On this basis, the authors designated a protein encoded in the *P*. *falciparum* genome (PF3D7_1417400), which contains putative cyclic nucleotide binding sites, as PfEpac [16].

An essential step in erythrocyte invasion by the malaria merozoite is the formation of a close association between the parasite and the erythrocyte surface known as the tight junction or moving junction, which rapidly expands to form a doughnut-shaped structure, through which the merozoite passes into the host cell [17]. Apical membrane antigen 1 (AMA1), a micronemal integral membrane protein that is discharged onto the merozoite surface just prior to invasion, is a key player in the formation of the tight junction. For this, the ectodomain of AMA1 forms adhesive interactions with rhoptry neck protein 2 (RON2), another parasite protein, which is secreted from a third set of secretory organelles called rhoptries into the erythrocyte membrane [18–21]. In addition to this crucial role of its ectodomain, the short cytoplasmic tail of AMA1 appears to play an indispensable signalling or sensing role in invasion, because AMA1 function is impaired by mutations that either remove the domain completely or that prevent phosphorylation of specific cytoplasmic tail residues (Ser_610_ or Thr_613_ in *P*. *falciparum*) [22–24]. More recent evidence suggests that this phosphorylation occurs in an ordered or hierarchical manner, with phosphorylation of Thr_613_ by glycogen synthase kinase 3 being dependent on prior phosphorylation of Ser_610_ by PKA [22,23]. However, genetic evidence for a role for parasite PKA in phosphorylation of the AMA1 cytoplasmic tail is lacking, and the structural consequences of its phosphorylation are unknown.

We recently reported that regulation of cAMP levels in asexual blood stage *P*. *falciparum* is governed by a dual-specific phosphodiesterase called phosphodiesterase beta (PDEβ) [25]. Conditional ablation of *PDEβ* led to a 70% reduction in invasion and increased phosphorylation of over 230 parasite protein phosphosites, most of which contained a minimal PKA consensus motif (R/K, x, pS/pT), suggesting that the *PDEβ* knockout phenotype resulted from inappropriate hyper-activation of PKA due to uncontrolled cAMP levels. Of note, phosphorylation of AMA1 Ser_610_ was up-regulated in the *PDEβ* null mutant, further supporting the notion that it is a PKA substrate [25]. In the present study, we have examined the role of cAMP signalling in *P*. *falciparum* blood stage development in detail. To do this, we targeted both ACβ, the only adenylyl cyclase expressed in the asexual blood stage parasite, and PKAc, the catalytic subunit of the parasite’s cAMP-dependent protein kinase. Conditional deletion of *ACβ* and *PKAc* allowed us to determine, respectively, the effects on the parasite of the absence of cAMP synthesis and of the absence of cAMP effector kinase activity. In both cases, gene ablation completely blocked merozoite invasion, demonstrating essential roles for ACβ and PKAc in this process. Deletion of *ACβ* also led to an unexpected delay in egress, suggesting a potential role for cAMP signalling in this cGMP-dependent process. We also showed that PfEpac is not required for parasite growth and thus cannot be an important regulator of the cAMP-dependent signalling that is critical for invasion. We identified cAMP- and PKA-dependent phosphorylation sites in many proteins associated with invasion, including AMA1, and showed that phosphorylation of AMA1 Ser_610_ leads to substantial structural changes in the protein’s cytoplasmic tail domain that may underlie the crucial signalling role of this protein in invasion.

## Results

### Generation of genetic tools to study the role of cAMP and its effector kinase, PKAc

Previous studies of cAMP signalling in *Plasmodium* have relied on the use of pharmacological tools originally developed for mammalian ACs and PKAs. However, the specificity of these compounds in highly evolutionarily divergent eukaryotes such as protozoan parasites is unclear. To investigate cAMP signalling in *P*. *falciparum* blood stages, we therefore generated two transgenic parasite lines designed to allow the conditional disruption of either *ACβ* or *PKAc*. Both lines were generated on the genetic background of *P*. *falciparum* parasites that stably express dimerisable Cre (DiCre), a split Cre-recombinase, the activity of which is induced in the presence of rapamycin (RAP) [26]. In each case, the target genes were ‘floxed’ such that treatment with RAP was expected to lead to excision of DNA sequences encoding the respective catalytic domains of the enzymes. At the same time, the genes were modified by fusion to sequences encoding a C-terminal triple hemagglutinin (HA_3_) epitope tag.

**Fig 1:**
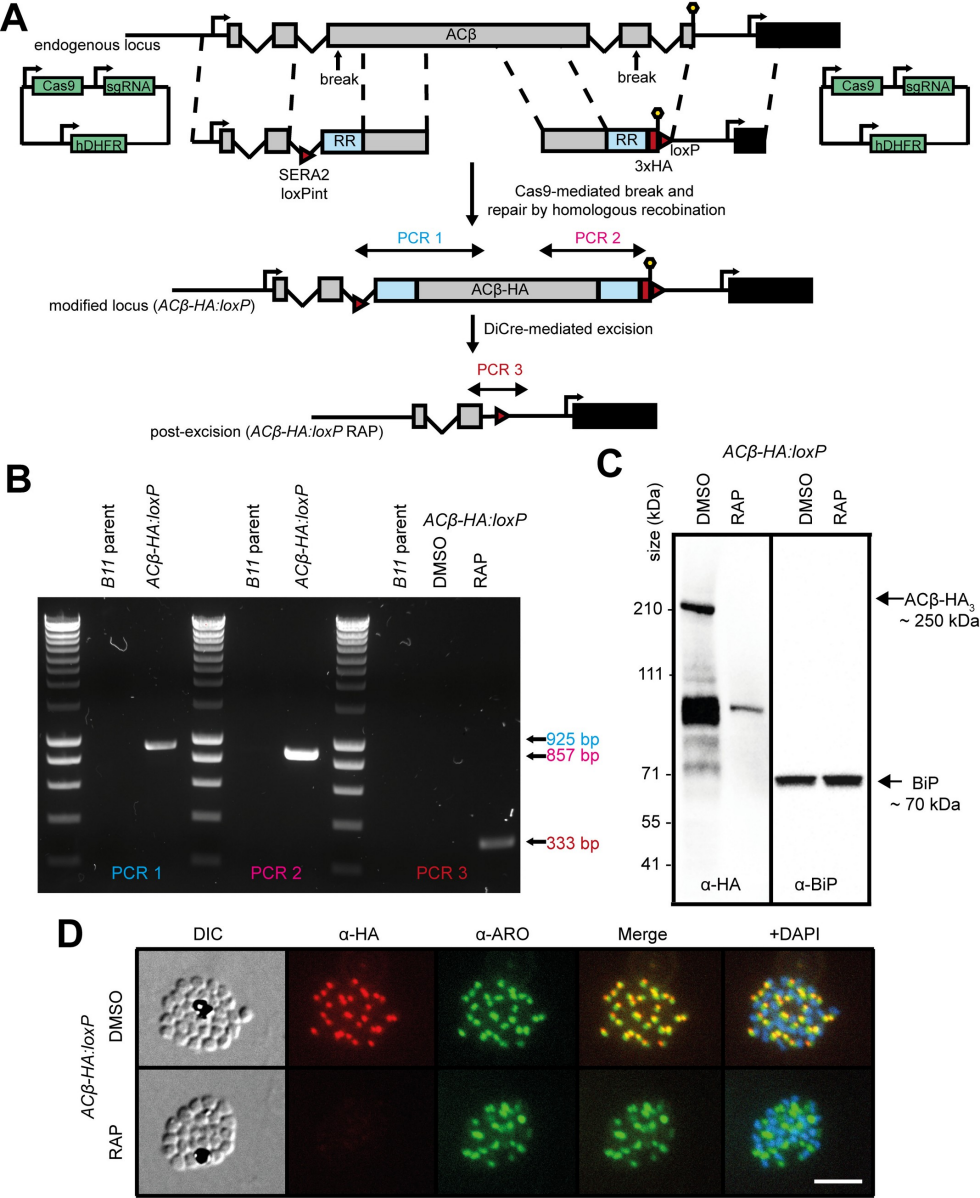
Conditional disruption of ACβ expression. (A) Schematic representation of the Cas9-mediated creation of the *ACβ-HA*:*loxP* line in DiCre-expressing *P*. *falciparum* parasites and subsequent RAP-induced deletion of the modified gene. Double-headed arrows represent the regions amplified by PCR in (B). Red arrowheads represent loxP sites, lollipops represent translational stop codons, black boxes indicate the position of an open reading frame downstream of ACB and light blue boxes indicate regions of re-codonised sequence. (B) Diagnostic PCR verifying successful integration of both repair constructs and successful genetic excision upon treatment with RAP in an *ACβ-HA*:*loxP* clone. Early rings (0–4 h post invasion) were treated with RAP or vehicle only (DMSO), and genomic DNA from schizonts (approximately 40 h posttreatment) was used in these PCRs. (C) Western blots confirming HA tagging and RAP-induced ablation of ACβ expression in the *ACβ-HA*:*loxP* line. In addition to a signal of the predicted intact ACβ molecular mass, bands of lower molecular mass were also detected, likely due to proteolysis. Antibodies to the ER protein BiP (PF3D7_0917900) were used as a loading control. (D) IFA showing localisation of ACβ-HA_3_ in close proximity to the rhoptry protein ARO (PF3D7_0414900) in schizonts, and ablation of expression by RAP treatment. Over 99% of all RAP-treated *ACβ-HA*:*loxP* schizonts examined by IFA were HA-negative in three independent experiments. Scale bar, 10 μm. ACβ, adenylyl cyclase beta; ARO, armadillo repeats only protein; BiP, binding immunoglobulin protein; Cas9, CRISPR-associated protein 9; DIC, differential interference contrast; DiCre, dimerisable Cre-recombinase; ER, endoplasmic reticulum; HA_3_, triple hemagglutinin; hDHFR, human dihydrofolate reductase selectable marker; IFA, immunofluorescence assay; loxPint, loxP containing intron; RAP, rapamycin; RR, recodonised-region; SERA2, serine repeat antigen 2; sgRNA, single guide RNA.

Generation of the *ACβ* conditional knockout line (*ACβ-HA*:*loxP)* was achieved in two steps using marker-free CRISPR-associated protein 9 (Cas9)-mediated genome editing (Fig 1A). The desired genetic modifications were verified by PCR (Fig 1B), and expression of tagged ACβ (ACβ-HA_3_) was confirmed by western blot using an anti-HA antibody (Fig 1C). Immunofluorescence assays (IFAs) demonstrated co-localisation of ACβ-HA_3_ in schizonts with the rhoptry-associated protein, armadillo repeats only protein (ARO) (Fig 1D), pointing to a rhoptry localisation for ACβ. This mirrors a recent report that localised *Toxoplasma gondii* ACβ to the rhoptry surface [27], but contrasts with previous suggestions of a cytoplasmic localisation for *P*. *falciparum* ACβ [16].

**Fig 2:**
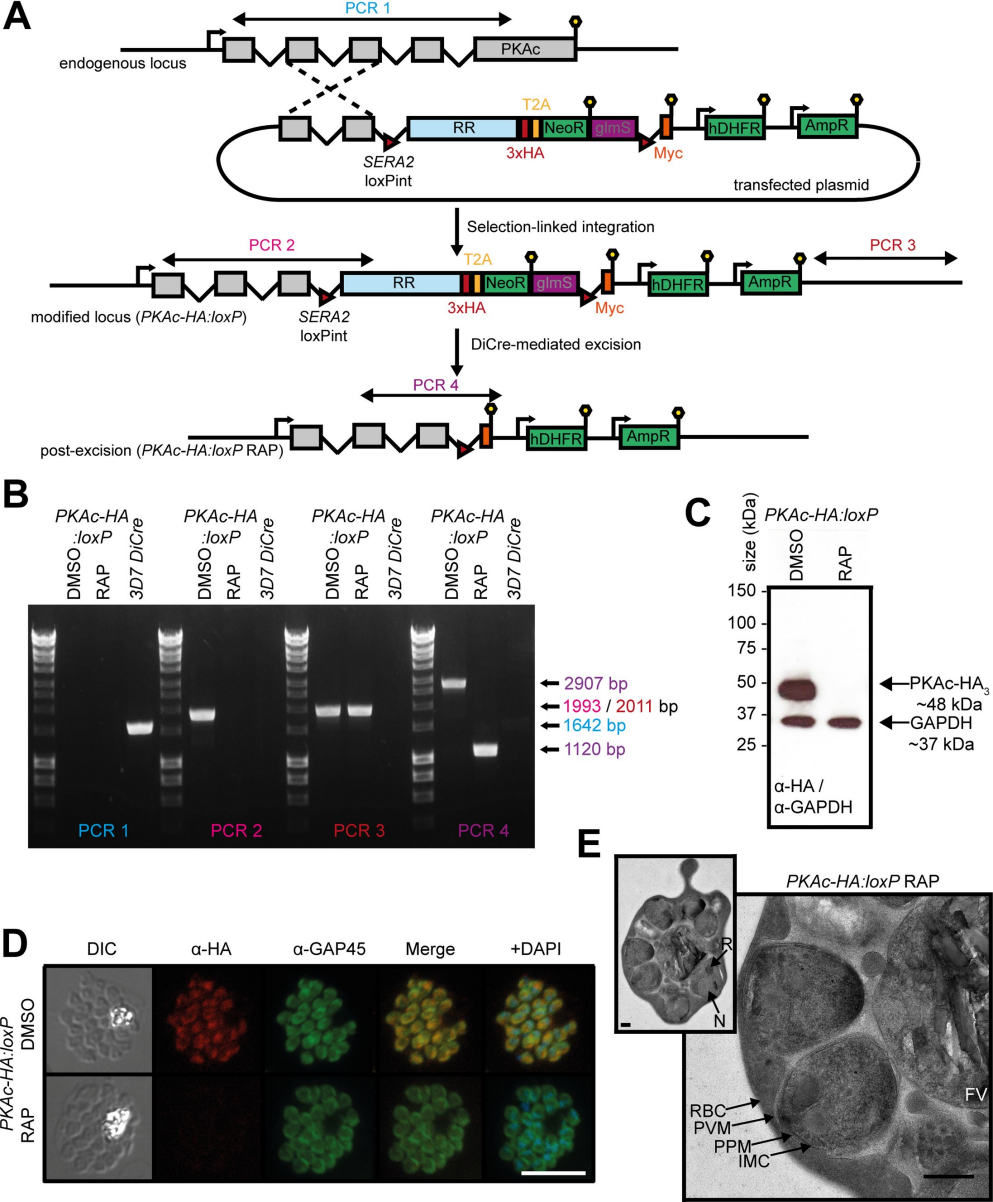
Conditional disruption of PKAc expression. (A) Schematic representation of the SLI strategy used to produce the *PKAc-HA*:*loxP* line and RAP-induced disruption of the gene. Double-headed arrows represent the regions amplified by PCR in (B). Red arrowheads represent loxP sites, lollipops represent translational stop codons, and light blue boxes indicate regions of re-codonised sequence. glmS was not exploited in these experiments. (B) Diagnostic PCR analysis verifying successful SLI to produce the *PKAc-HA*:*loxP* line and successful excision of floxed sequences upon treatment with RAP. Rings (about 20 h post invasion) were RAP or DMSO treated for 2 h, and genomic DNA from schizonts (about 20 h post-treatment) was used in these PCRs. (C) Western blots showing expression (DMSO) and ablation (RAP) of PKAc-HA_3_ in *PKAc-HA*:*loxP* parasites. Expression of GAPDH (PF3D7_1462800) is shown as a loading control. (D) IFA showing the diffuse localisation of PKAc-HA_3_ (DMSO) and the loss of expression upon RAP treatment. Over 99% of all RAP-treated *PKAc-HA*:*loxP* schizonts examined by IFA were HA-negative in three independent experiments. (E) Electron micrograph of a segmented RAP-treated *PKAc-HA*:*loxP* schizont from high-pressure frozen, freeze-substituted plastic sections. Inset: image of an entire *PKAc-HA*:*loxP* schizont showing the typical morphology of a mature schizont prior to PVM rupture. Main image: a more detailed view of two of the merozoites within the schizont. Scale bar, 500 nm. AmpR, ampicillin resistance cassette used for plasmid selection in bacteria; DIC, differential interference contrast; DiCre, dimerisable Cre-recombinase; FV, food vacuole; GAPDH, glyceraldehyde 3-phosphate dehydrogenase; GAP45, glideosome-associated protein 45; glmS, glucosamine-6-phosphate riboswitch ribozyme; HA_3_, triple hemagglutinin; hDHFR, human dihydrofolate reductase selectable marker; IFA, immunofluorescence assay; IMC, inner membrane complex; loxPint, loxP containing intron; Myc, c-myc tag; N, nucleus; NeoR, neomycin resistance selectable marker; PKAc, catalytic subunit of cAMP-dependent protein kinase; PPM, parasite plasma membrane; PVM, parasitophorous vacuole membrane; R, rhoptries; RAP, rapamycin; RBC, red blood cell membrane; RR, recodonised-region; *SERA2*, serine repeat antigen 2 gene; SLI, selection-linked integration; T2A, thosea asigna virus 2A peptide.

To generate a PKAc conditional knockout line (called *PKAc-HA*:*loxP*), we exploited the recently developed selection-linked integration (SLI) method [28] (Fig 2A). Successful modification of the *PKAc* gene was verified by PCR (Fig 2B), and expression of tagged PKAc-HA_3_ in the *PKAc-HA*:*loxP* parasites was confirmed by western blot (Fig 2C). Examination of the transgenic parasites by IFA (Fig 2D) revealed a diffuse HA-specific signal that encompassed the parasite cytosol as delineated by co-staining with the inner membrane complex marker glideosome-associated protein 45 (GAP45). A cytosolic location for PKAc-HA_3_ was confirmed by subcellular fractionation of parasite extracts produced by hypotonic lysis (S1A Fig).

### Both *ACβ* and *PKAc* are essential for parasite proliferation

**Fig 3:**
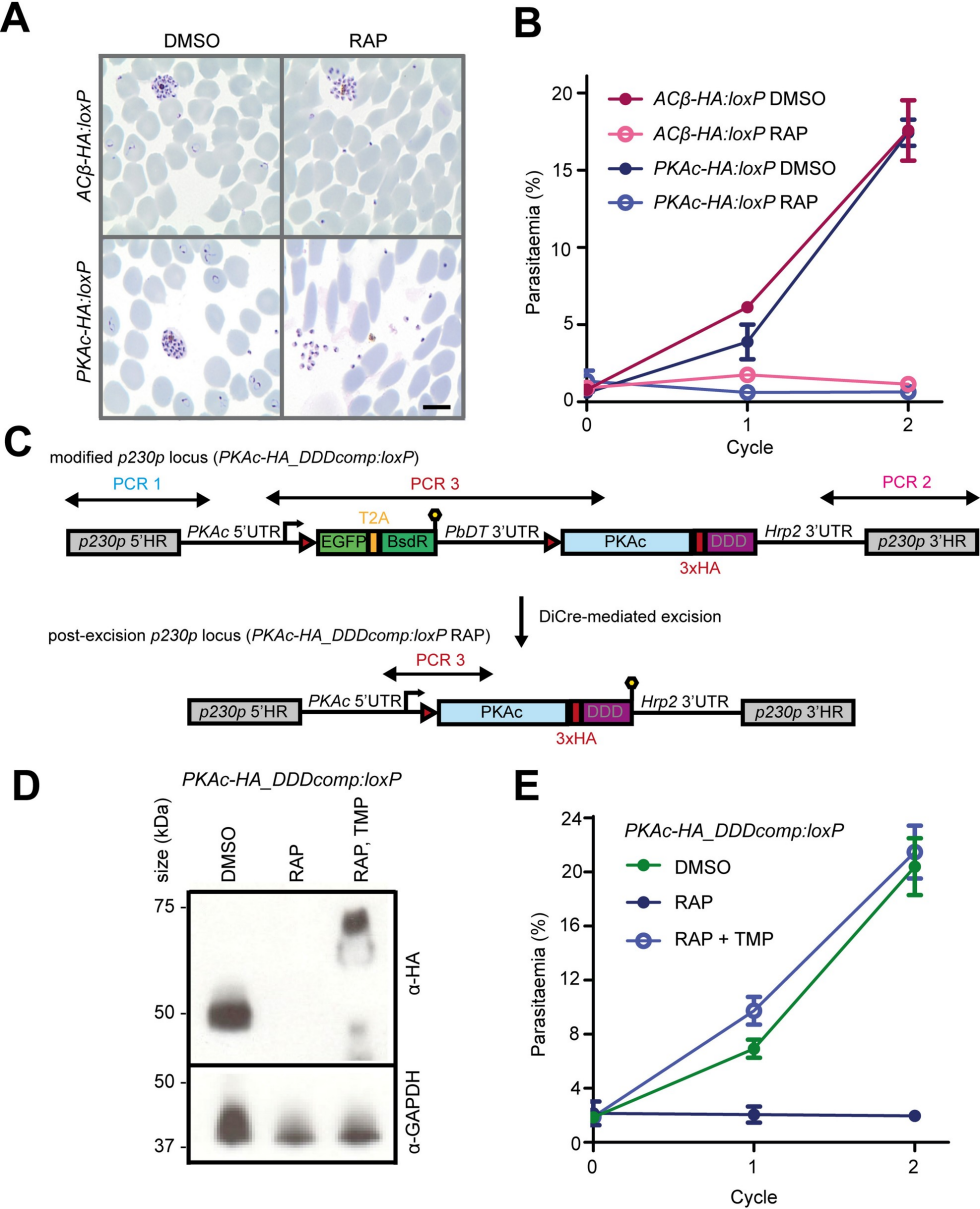
ACβ and PKA are both essential for parasite proliferation. (A) Giemsa-stained blood films showing ring-stage parasites following egress of DMSO-treated *ACβ-HA*:*loxP* and *PKAc-HA*:*loxP* parasites (left) and the absence of rings following egress of RAP-treated parasites. Scale bar, 5 μm. (B) Growth curves showing changes in parasitaemia of *ACβ-HA*:*loxP* and *PKAc-HA*:*loxP* parasites treated with DMSO (vehicle only control) or RAP. Means from three replicates are plotted. Error bars, SD. (C) Schematic representation of the approach used to genetically complement the *PKAc-HA*:*loxP* line by Cas9-mediated introduction of a RAP-inducible, trimethoprim (TMP)-stabilised HA-tagged PKAc transgene at the *p230p* locus to create the *PKAc-HA_DDDcomp*:*loxP* line. Double-headed arrows represent the regions amplified by PCR in S4C Fig. Red arrowheads represent loxP sites, lollipops represent translational stop codons, and light blue boxes indicate regions of re-codonised sequence. (D) Western blots showing the RAP-inducible switch from expression of PKAc-HA_3_ from the *PKAc* locus to expression of TMP-stabilised PKAc-HA_3__DDD from the *P230p* locus. Note the decreased mobility of the PKAc-HA_3__DDD resulting from its fusion to the DDD. GAPDH is shown as a loading control. Some lower molecular weight products are detected in the RAP TMP lane, likely due to incomplete stabilisation of all protein species. (E) Growth curve showing rescue of growth of PKAc-HA_3_–deficient parasites by TMP-mediated stabilisation of PKAc-HA_3__DDD. Means from three replicates are plotted. Error bars, SD. Data associated with this figure can be found in the supplemental data file (S1 Data). ACβ, adenylyl cyclase beta; BsdR, blasticidin resistance selectable marker; Cas9, CRISPR associated protein 9; DDD, DHFR degradation domain; DHFR, dihydrofolate reductase; EGFP, enhanced green fluorescent protein; GAPDH, glyceraldehyde 3-phosphate dehydrogenase; HA_3_, triple hemagglutinin; PKA, cAMP-dependent protein kinase; PKAc, catalytic subunit of cAMP-dependent protein kinase; RAP, rapamycin; TMP, trimethoprim.

To investigate the essentiality of ACβ and PKAc, highly synchronised ring-stage cultures of each line were treated with RAP to induce excision of sequences encoding the catalytic domains of each enzyme. DiCre-mediated gene excision was reproducibly highly efficient (Fig 1D and Fig 2D). RAP-treated *ACβ-HA*:*loxP* and *PKAc-HA*:*loxP* rings developed normally to mature schizonts in the erythrocytic cycle of treatment (cycle 0) (Figs 1D and 2D and Fig 2E), and in both cases these schizonts ruptured and released merozoites. However, no new ring-stage parasites were observed in the ACβ-null and PKAc-null cultures at the beginning of the next erythrocytic cycle following treatment (cycle 1) (Fig 3A). Consistent with this, using flow cytometry–based analysis, we observed complete arrest of parasite expansion beyond cycle 0 in the RAP-treated cultures (Fig 3B and S1B Fig), indicating that both genes are essential for parasite survival.

To determine whether the phenotype observed upon RAP treatment of the *PKAc-HA*:*loxP* line was a direct result of loss of PKAc function, we used a genetic complementation approach to rescue the lethal phenotype. For this, we further modified the *PKAc-HA*:*loxP* line to integrate a second copy of the *PKAc-HA*_*3*_ gene into the genomic *p230p* locus (Fig 3C and S1D Fig). This gene was additionally fused to a dihydrofolate reductase destabilisation domain (DDD) and placed downstream of a floxed promoter sequence. RAP treatment of the resulting parasite line (called *PKAc-HA_DDDcomp*:*loxP*) was expected to excise the floxed endogenous *PKAc-HA*_*3*_ locus whilst simultaneously inducing expression of the second, *PKAc-HA*_*3*_*-DDD* gene, which could be stabilised by the additional presence of trimethoprim (TMP) (Fig 3D and S1C Fig). Growth of RAP-treated *PKAc-HA_DDDcomp*:*loxP* parasites was only observed in the presence of TMP, and these parasites proliferated at a rate comparable to that of DMSO-treated control *PKAc-HA_DDDcomp*:*loxP* parasites (Fig 3E). This indicated successful conditional genetic complementation of the PKAc-null mutant and confirmed the essentiality of the *PKAc* gene.

### Egress does not require PKAc but is delayed in the absence of cAMP synthesis

**Fig 4:**
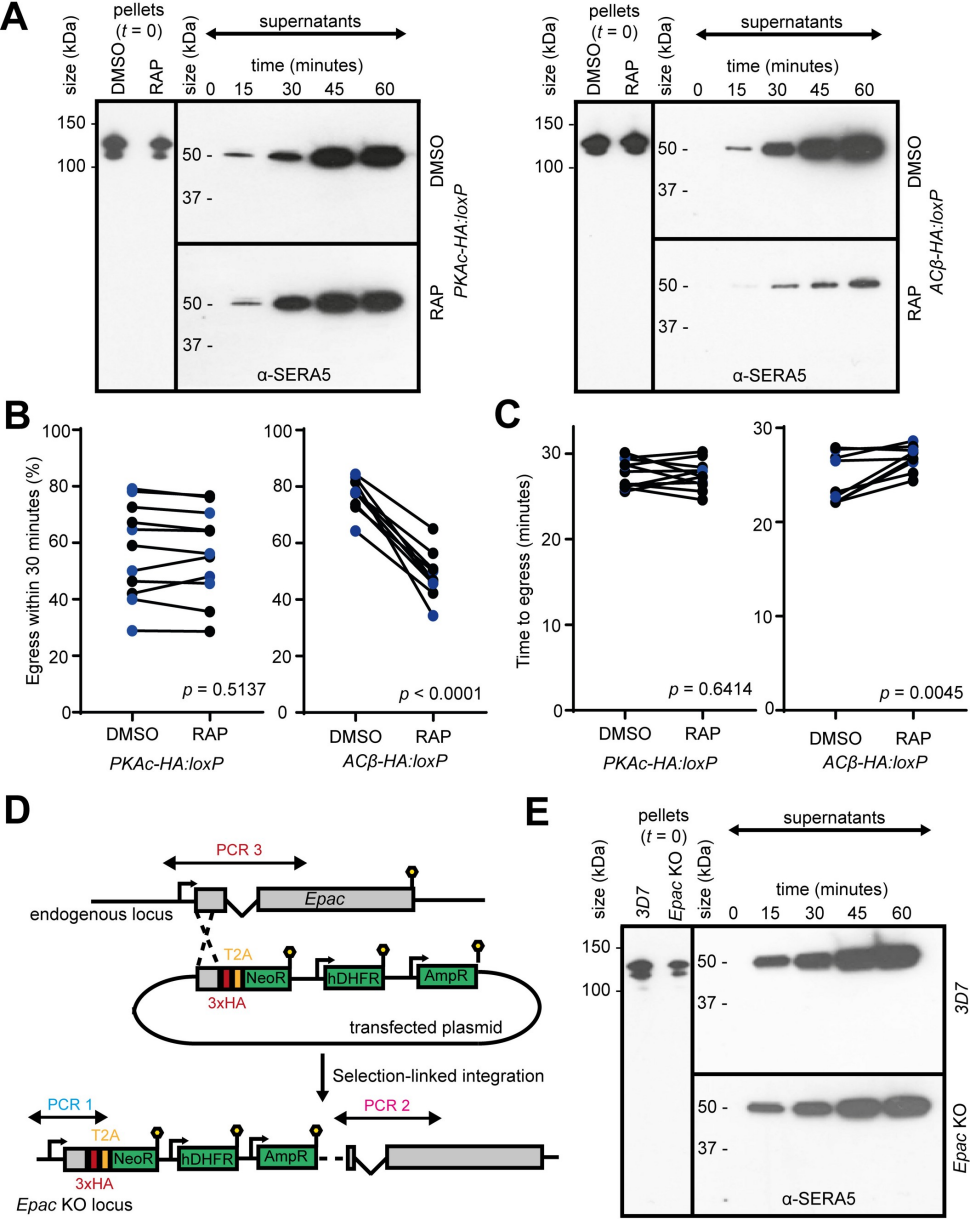
PKA, cAMP, and Epac are not required for egress. (A) Western blots data monitoring egress kinetics of DMSO- and RAP-treated *PKAc-HA*:*loxP* and *ACβ-HA*:*loxP* schizonts. The slower onset of detection of SERA5 p50 in RAP-treated *ACβ-HA*:*loxP* parasites indicates delayed or impaired egress in the absence of cAMP. Blots are representative of two biological repeats, which are both quantified in S4A Fig. (B) Quantification of the proportion of schizonts rupturing in 30-min videos of DMSO- and RAP-treated parasites. For each video, one parasite population (DMSO or RAP) was stained with Hoechst (indicated in blue on the plots). The *p*-values derive from paired *t* tests. (C) Quantification of the mean time taken for the DMSO- and RAP-treated parasites from (B) to progress to egress, as measured by visual analysis of the same video microscopy data shown in panel (B). The *p*-values derive from paired *t* tests. For all data in (B) and (C), each point is the mean for one population (DMSO or RAP) from a single video (50–100+ schizonts). Ten videos were quantified from at least three independent experiments. (D) Schematic representation of the selection-linked targeted homologous recombination-based approach used to disrupt the PfEpac gene. Lollipops represent translational stop codons. Validation of this line by PCR is shown in S1E Fig. (E) Western blot data indicating normal rupture of PfEpac-deficient schizonts. Data associated with this figure can be found in the supplemental data file (S1 Data). AmpR, ampicillin resistance cassette used for plasmid selection in bacteria; cAMP, cyclic AMP; Epac, exchange protein directly activated by cAMP; hDHFR, human dihydrofolate resistance selectable marker; KO, knockout; NeoR, neomycin resistance selectable marker; PKA, cAMP-dependent protein kinase; p50, processed 50 kDa form; RAP, rapamycin; SERA5, serine repeat antigen 5; T2A, thosea asigna virus 2A peptide.

Whilst our initial observations indicated an important role for ACβ and PKAc in erythrocyte invasion, we next sought to examine whether cAMP signalling also contributes to egress. To do this, we compared the kinetics of egress of preparations of highly synchronous mature DMSO- and RAP-treated *PKAc-HA*:*loxP* or *ACβ-HA*:*loxP* schizonts by monitoring the appearance over time of proteolytically processed forms of the abundant PV protein serine repeat antigen 5 (SERA5) in schizont culture supernatants [29]. As shown in Fig 4A, no differences were observed between the rates of egress of control and RAP-treated *PKAc-HA*:*loxP* schizonts. In contrast, we observed a marked reduction in the rate of progress to egress in RAP-treated *ACβ-HA*:*loxP* schizonts compared with their DMSO-treated counterparts (Fig 4A). These findings were confirmed by time-lapse video microscopy (Fig 4B and Fig 4C, S1 and S2 Movies). Collectively, these results pointed to an unexpected PKA-independent role for cAMP in the fine-tuning of egress kinetics.

In view of this finding, we investigated whether another protein, independent of PKA, might respond to cAMP and activate pathways that modulate egress. Besides PKG and PKAr, the only other molecule encoded by the *P*. *falciparum* genome predicted to possess cyclic nucleotide binding domains is one previously designated PfEpac (encoded by the PF3D7_1417400 gene), which has been suggested to be a modulator of calcium release (a prerequisite for egress) [16]. To investigate the importance of PfEpac in parasite viability, we used an SLI-based approach to directly disrupt the *PfEpac* gene (Fig 4D). The resulting PfEpac-null line was validated by PCR, western blot, and IFA (S1E–S1G Fig). The mutant parasites displayed no impairment of growth (S1H Fig) and no change in the kinetics of egress compared with the parental parasite line (Fig 4E). These results are fully in accord with previous evidence that *PfEpac* is dispensable in in vitro culture [30,31] and indicate that PfEpac is not a regulator of parasite egress.

### cAMP and PKAc are both critical for invasion

**Fig 5:**
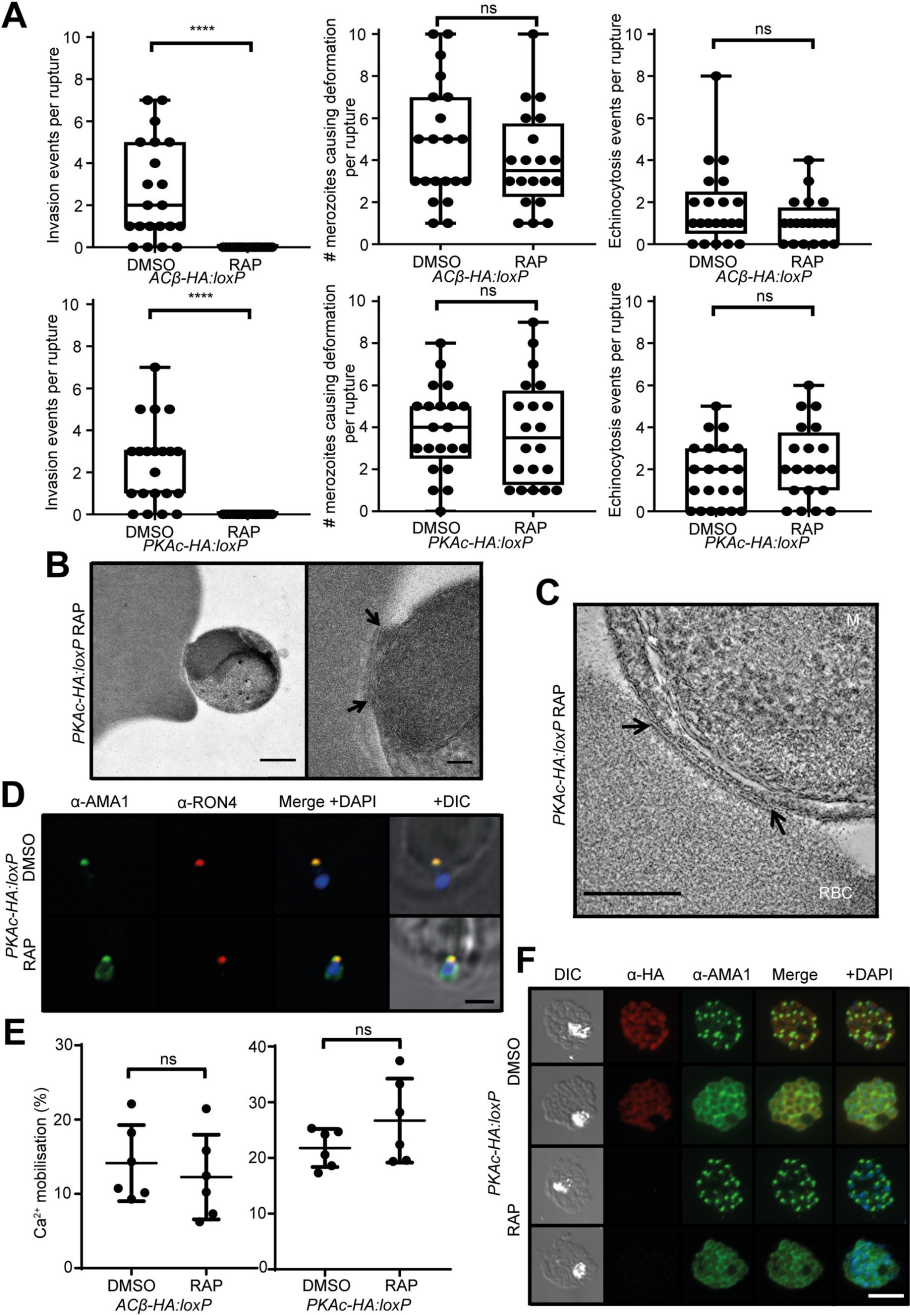
Invasion is critically dependent on cAMP and PKAc, but calcium mobilisation and microneme secretion are not. (A) Quantification of invasion, merozoite-induced erythrocyte surface deformation, and echinocytosis observed by video microscopy following rupture of DMSO- and RAP-treated *PKAc-HA*:*loxP* and *ACβ-HA*:*loxP* schizonts. At least 20 videos per condition were quantified. Statistical significance was assessed by *t* test; ns indicates not significant (*p* > 0.05), whereas **** indicates *p* < 0.0001. (B) Electron micrograph of a RAP-treated *PKAc-HA*:*loxP* merozoite attached to the surface of an erythrocyte from high-pressure frozen, freeze-substituted plastic sections. Left: apical attachment of a merozoite to the surface of the erythrocyte. Scale bar, 500 nm. Right: a more detailed view of the electron-dense attachment region (arrowed) showing the close association of the apical end of the parasite and the erythrocyte membrane. Scale bar, 100 nm. (C) Electron tomography of the attachment region between a RAP-treated *PKAc-HA*:*loxP* merozoite and the RBC surface. Image is a sum of 30 central sections from the tomogram. Arrows indicate a region of apparent thickening of the RBC membrane. Full-tilt series in S7 Movie. Scale bar, 250 nm. (D) Super-resolution immunofluorescence imaging of *PKAc-HA*:*loxP* merozoites attached to the RBC surface. For the DMSO-treated group, parasites’ medium contained 1 μM cyotchalasin D to arrest invasion at tight junction formation. Additional images are shown in S3 Fig. Scale bar, 2 μm. (E) Induction of calcium mobilisation using 100 μM zaprinast in synchronous Fluo-4–loaded late-stage schizonts assayed by fluorimetry. The signal was normalised to DMSO carrier (0% signal) and 20 μM A23187 ionophore (100% signal). Means from six technical replicates (three samples from two biological replicates) are plotted. Error bars, SD. (F) IFA showing re-localisation of AMA1 from micronemes to the merozoite periphery in DMSO- and RAP-treated *PKAc-HA*:*loxP* schizonts. Quantification of 100 imaged schizonts from three individual biological replicates indicated no significant difference between peripheral and punctate staining of AMA1 between the two treatments (DMSO 55.88% ± 2.25% punctate, 41.21% ± 2.23% peripheral, and RAP 55.86% ± 2.93% punctate, 41.14% ± 1.93% peripheral). IFA analysis was performed on highly synchronous cultures that were treated with 20 μM E64 approximately 44 h post invasion for approximately 4 h. Scale bars, 5 μm. Data associated with this figure can be found in the supplemental data file (S1 Data). AMA1, apical membrane antigen 1; cAMP, cyclic AMP; DIC, differential interference contrast; E64, cysteine protease inhibitor; IFA, immunofluorescence assay; M, merozoite; ns, not significant; PKAc, catalytic subunit of cAMP-dependent protein kinase; RAP, rapamycin; RBC, red blood cell.

The above results showed that cAMP- or PKA-deficient parasites are able to form schizonts that undergo egress yet are unable to proliferate further in culture. The absence of ring-stage parasites in cycle 1 following RAP treatment of *ACβ-HA*:*loxP* or *PKAc-HA*:*loxP* parasites indicated a selective defect in host erythrocyte invasion (Fig 3A). This was confirmed using a flow cytometry–based assay that showed that merozoites released from the RAP-treated cultures are able to bind to fresh host erythrocytes but do not invade them to form rings or trophozoites (S2A Fig). To examine this invasion deficit in more detail, we examined the behaviour of naturally released ACβ-null and PKAc-null merozoites by live video microscopy. No successful invasion events were observed following the rupture of at least 20 schizonts from each RAP-treated line (Fig 5A, S3–S6 Movies). However, the visual analysis showed that the free merozoites were able to transiently interact with and deform host erythrocytes with a frequency comparable to that observed in their DMSO-treated counterparts, suggesting that the parasite actinomyosin motor is active in the absence of cAMP or PKAc [32,33]. We also observed similar numbers of echinocytosis events induced by merozoites released from the DMSO- and RAP-treated *ACβ-HA*:*loxP* and *PKAc-HA*:*loxP* cultures, in which targeted host erythrocytes appear to transiently shrink and become ‘spiky’ following contact with the merozoites (Fig 5A, S3–S6 Movies). Induction of echinocytosis by *P*. *falciparum* merozoites is associated with rhoptry discharge [32], so our observations suggest that rhoptry discharge is independent of cAMP levels or PKAc activity.

The first irreversible step in invasion is the formation of the ‘tight junction’, mediated primarily by associations between merozoite surface AMA1 with RON proteins delivered from the rhoptries into the erythrocyte membrane [17–21]. Using transmission electron microscopy (TEM), we analysed thin sections in which mature RAP-treated *PKAc-HA*:*loxP* schizonts were allowed to rupture in the presence of erythrocytes. We observed intact schizonts (Fig 2E), recently ruptured schizonts, free merozoites, and merozoites attached to the erythrocyte surface, but we did not observe any merozoites arrested at later stages of invasion. Detailed analyses by electron tomography showed the presence of a more electron-dense zone of the red blood cell (RBC) membrane at the attachment site (Fig 5C and S7 Movie), a feature consistent with previous observations describing tight junction formation [34,35]. Super-resolution immunofluorescence imaging detected punctate zones of co-localisation of AMA1 and RON4 at apical attachment sites of DMSO- and RAP-treated *PKAc-HA*:*loxP* merozoites bound to erythrocytes (Fig 5D and S3 Fig).

Taken together, our data indicate that the invasion defect observed in the absence of cAMP or PKAc occurs at a late stage of the pathway; mutant merozoites are able to associate with erythrocytes, secrete invasion-related proteins, exert force upon and induce physical changes in prospective host cells, but still fail to complete invasion.

### Calcium mobilisation, microneme discharge, and rhoptry secretion are independent of cAMP and PKAc

To better understand the molecular basis of the invasion defect observed in *ACβ*- and *PKAc*-null parasites, we assessed whether key processes known to occur upstream of invasion are affected by the absence of cAMP or PKA. We first investigated calcium signalling and microneme release, as both processes have been reported to be cAMP dependent in merozoites exposed to K^+^ concentrations mimicking extracellular conditions [16]. For this we used the cell-permeable calcium-sensitive fluorophore Fluo-4-AM, as previously described [8], to measure calcium flux in *ACβ-HA*:*loxP* or *PKAc-HA*:*loxP* mature schizonts upon treatment with the phosphodiesterase inhibitor zaprinast. No significant differences were detected between RAP-treated parasites and DMSO-treated controls (Fig 5E). We next used IFA to examine re-localisation of the microneme-resident protein AMA1 onto the surface of intracellular merozoites [2] as a proxy for microneme discharge. Again, visual quantitation of the proportion of schizonts displaying peripheral AMA1 staining in populations of DMSO- and RAP-treated *ACβ-HA*:*loxP* or *PKAc-HA*:*loxP* parasites revealed no differences in the efficiency of AMA1 discharge (Fig 5F and S2B Fig), indicating that AMA1 is efficiently secreted from micronemes in the absence of PKAc and cAMP.

**Fig 6:**
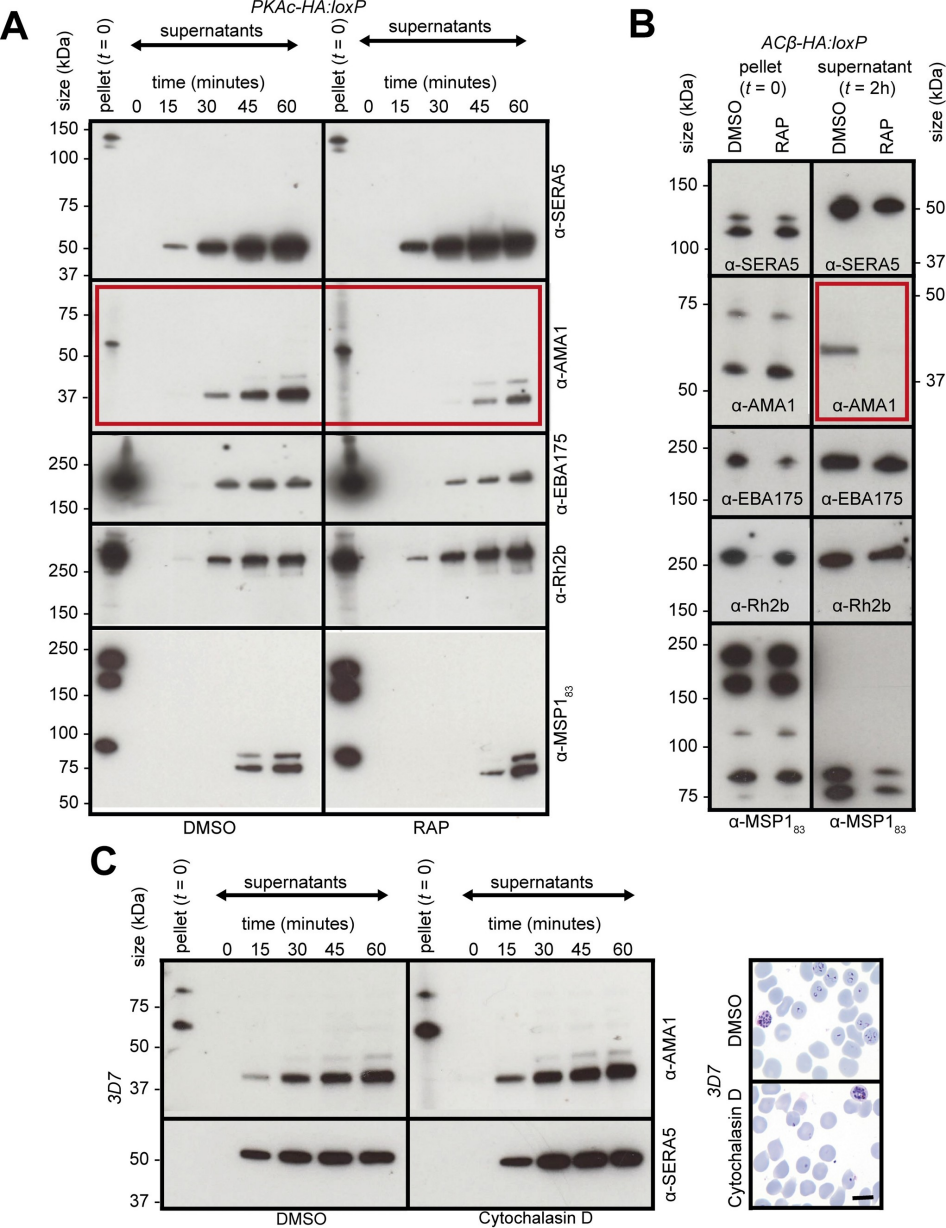
Efficient surface shedding of AMA1 requires cAMP and PKA. (A) Western blot of culture supernatants from a time course of egressing DMSO- and RAP-treated *PKAc-HA*:*loxP* parasite cultures. Progression of egress is indicated by detection of the SERA5 p50 fragment. Shedding of surface adhesins AMA1, EBA175, Rh2b, and MSP1 are monitored by their detection in supernatants using the indicated antibodies. For each western blot, a representative image from one of three independent experiments is shown. The full-length blots used to produce this figure are shown in S4B Fig. Densitometry analyses of three biological replicates are shown in S4C Fig and S4D Fig. No significant differences in EBA175 shedding were observed after one hour, but 4.2 ± 1.6-fold less AMA1 was shed in RAP- compared with DMSO-treated *PKAc-HA*:*loxP* parasites. Blots are representative of three biological repeats. (B) Western blots indicating the presence of SERA5 p50 and shed surface adhesins in supernatants from egressing DMSO- and RAP-treated *ACβ-HA*:*loxP* parasites. A single time point was used because the RAP-treated population are slower to egress. The full-length blots used to produce this figure are shown in S4B Fig. Blots shown are representative of two biological repeats. (C) Left: western blot of culture supernatants from a time course of egressing 3D7 parasites showing unaltered AMA1-shedding kinetics in the presence or absence of the invasion inhibitor cytochalasin D (1 μM). Right: Giemsa-stained blood films confirming the cytochalasin D–mediated block in invasion by an absence of ring-stage parasites in the treated cultures. Scale bar, 5 μm. AMA1, apical membrane antigen 1; cAMP, cyclic AMP; EBA175, erythrocyte binding antigen 175; PKA, cAMP-dependent protein kinase; p50, processed 50 kDa form; RAP, rapamycin; Rh2b, reticulocyte binding protein homologue 2b; SERA5, serine repeat antigen 5.

As a further means of evaluating secretory organelle discharge in the mutant merozoites, we investigated the shedding of invasion-related molecules from the surface of egressed merozoites by western blot analysis of cell culture supernatants. As shown in Fig 6A, levels of the micronemal adhesin erythrocyte binding antigen 175 (EBA175) and the rhoptry-derived protein reticulocyte binding protein homologue 2b (Rh2b) shed over time from DMSO- and RAP-treated *PKAc-HA*:*loxP* parasites were indistinguishable. In contrast, we observed a significant reduction in levels of shed AMA1 from RAP- treated *PKAc-HA*:*loxP* or *ACβ-HA*:*loxP* merozoites (Fig 6A and Fig 6B). This reduction in AMA1 shedding was not a result of the inability of the mutant merozoites to invade erythrocytes, as shedding of AMA1 from wild-type 3D7 merozoites was unaffected by the presence of cytochalasin D, which inhibits invasion by blocking the activity of the parasite actinomyosin motor [32,36] (Fig 6C). Consistent with these observations, we only observed AMA1 at the apical end of DMSO-treated *PKAc-HA*:*loxP* merozoites attached to the erythrocyte surface, whilst attached PKAc-deficient merozoites retained detectable levels of AMA1 all around the merozoite periphery (Fig 5D and S3 Fig). Taken together, these results suggest that cAMP and PKA are not required for the secretion of invasion-related organelles but may play specific roles in the proteolytic shedding of AMA1.

### Phosphoproteomic profiling demonstrates cAMP and PKA-dependent phosphorylation of invasion-related proteins

**Fig 7:**
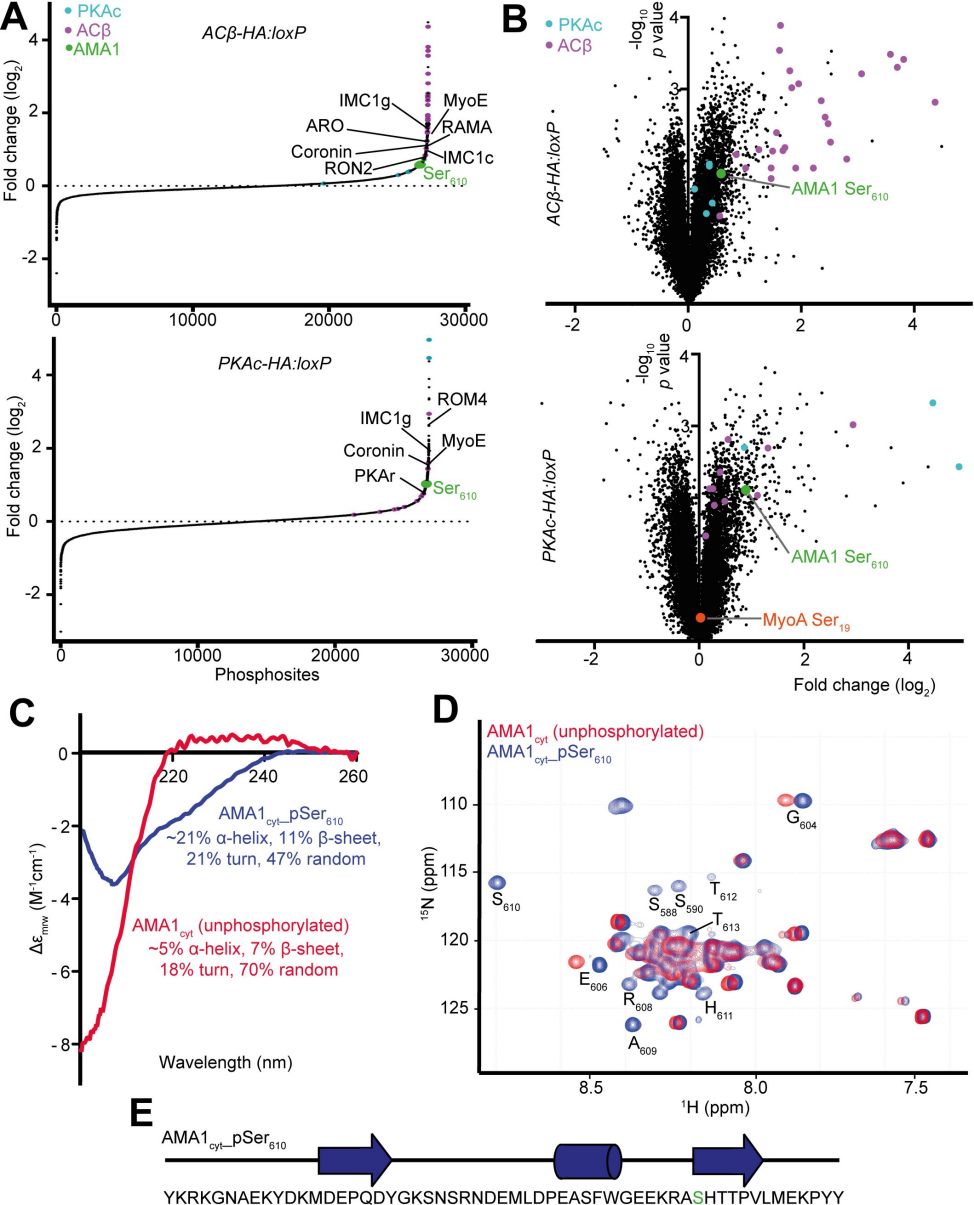
cAMP- and PKA-dependent phosphorylation of invasion-related proteins and induction of a structural change in the cytoplasmic tail of AMA1 by Ser_610_ phosphorylation. (A) S-curves representing the phosphosites detected by mass spectrometry in DMSO- versus RAP-treated *ACβ-HA*:*loxP* and *PKAc-HA*:*loxP* schizont/merozoite preparations. The most enriched site in each of the indicated invasion-related proteins is labelled. Data shown are from three technical triplicates and are representative of three biological repeats. (B) Volcano plots showing the changes in detection of phosphosites between DMSO- and RAP-treated *ACβ-HA*:*loxP* and *PKAc-HA*:*loxP*. The negative log_10_ transform of the *p*-value–derived Welch-corrected *t* test comparing three DMSO- and three RAP-treated replicates is plotted against the log_2_-transformed fold change in reporter ion intensity (DMSO/RAP). Significantly altered sites (*p* < 0.05) in the ACβ and PKAc proteins are indicated in magenta and blue, respectively. Data shown are from the three technical triplicates shown in panel (A) and are representative of three biological repeats. (C) Circular dichroism (CD) spectra of recombinant AMA1_cyt_ with and without treatment with recombinant mammalian PKA. (D) Overlay of a selected region of a 2D ^1^H-^15^N HSQC NMR spectra of unphosphorylated AMA1_cyt_ (red) and AMA1_cyt_ _pSer_610_ (blue). The residues are indicated below their positions on the AMA1_cyt__pSer_610_ spectra. Phosphorylation induced changes in the amide chemical shifts of the targeted Ser_610_ as well as surrounding residues. (E) Secondary structure prediction of the AMA1 cytoplasmic tail sequence (Ser_610_ indicated in green) as calculated by TALOS+ and Chemical Shift Index compared with random coil based on the ^1^H, ^15^N, ^13^C chemical shifts of AMA1_cyt_ _pSer_610_. Extended conformation is presented as arrows and alpha-helical conformation as a cylinder. No secondary structure elements are predicted for unphosphorylated AMA1_cyt_. Data associated with this figure can be found in the supplemental data file (S1 Data) and supporting table 1 (S1 Table). ACβ, adenylyl cyclase beta; AMA1, apical membrane antigen 1; AMA1_cyt_, AMA1 cytosolic domain; ARO, armadillo repeats only protein; cAMP, cyclic AMP; CD, circular dichroism; HSQC, heteronuclear single quantum coherence; IMC, inner membrane complex; MyoA, myosin A; MyoE, myosin E; NMR, nuclear magnetic resonance; PKA, cAMP-dependent protein kinase; PKAc, catalytic subunit of cAMP-dependent protein kinase; PKAr, regulatory subunit of cAMP-dependent protein kinase; RAMA, rhoptry-associated membrane antigen; RAP, rapamycin; ROM4, rhomboid protease 4; RON2, rhoptry neck protein 2; TALOS+, prediction of protein backbone and sidechain torsion angles from NMR chemical shifts program.

To gain further insight into the mechanisms through which cAMP and PKA control invasion, we profiled the ACβ- and PKA-dependent phosphoproteomes from parasite cultures comprising mature schizonts and merozoites—the parasite life stages in which cAMP-dependent signalling could plausibly exert control over the invasion process. Phosphopeptides enriched from trypsin-digested protein extracts of DMSO- and RAP-treated *ACβ-HA*:*loxP* and *PKAc-HA*:*loxP* parasites were examined by tandem mass spectrometry using isobaric labelling for quantification. We quantified over 20,000 different phosphorylation sites across the samples (Fig 7A, Fig 7B and S2C Fig), and comparison of DMSO-treated controls with their RAP-treated counterparts identified sites for each line that were enriched in the DMSO-treated controls. Of these sites, many were enriched in both *ACβ-HA*:*loxP* and *PKAc-HA*:*loxP* controls compared with their corresponding RAP-treated sample, indicating a dependence upon cAMP and PKA (S1 Table). Consistent with this, motif analysis showed that the phosphorylation sites fit a PKA consensus and closely resemble those identified in a recent study of sites enriched in a *P*. *falciparum* PDEβ knockout in which PKA activity was enhanced due to raised cAMP levels [25] (S2C Fig and S2D Fig). A total of 77 sites that we identified as being significantly hypophosphorylated in both the ACβ- and PKA-deficient parasites were also quantified in the recent PDEβ-dependent phosphoproteome. Most of these sites were significantly hyperphosphorylated in PDEβ-null parasites, evidence for regulation by cAMP-dependent signalling in three separate conditional knockout parasite lines. On this basis, we define a list of 61 sites in 39 proteins that are high-confidence targets of cAMP-dependent phosphorylation (Table 1). Of these sites, 42 possess a minimal consensus PKA substrate motif (R/K, x, pS/pT), suggesting that they could be phosphorylated directly by PKA. However, further work will be needed to determine whether these sites are directly phosphorylated in the parasite by PKA or another cAMP-regulated kinase.

**Table 1:** List of high-confidence targets of cAMP-dependent phosphorylation common to *ACβ*-, *PKA*-, and *PDEβ*-dependent phosphoproteome analyses.

Gene	Gene product	Sites
**PF3D7_0104300**	ubiquitin carboxyl-terminal hydrolase 1, putative	S2578
**PF3D7_0303200**	HAD superfamily protein, putative	S391
**PF3D7_0407800**	conserved *Plasmodium* protein, unknown function	S120, S379, S592, T645, S685, S688
**PF3D7_0418600**	regulator of chromosome condensation, putative	S1526
**PF3D7_0506900**	rhomboid protease ROM4	S191, T266
**PF3D7_0508900**	conserved *Plasmodium* protein, unknown function	S1741
**PF3D7_0517400**	FACT complex subunit SPT16, putative	S566
**PF3D7_0525800**	inner membrane complex protein 1g, putative	S267, S268, S270
**PF3D7_0609800**	palmitoyltransferase DHHC2, putative	S277
**PF3D7_0610400**	histone H3	S29, S33
**PF3D7_0618000**	conserved *Plasmodium* membrane protein, unknown function	S211
**PF3D7_0706500**	conserved *Plasmodium* protein, unknown function	S650
**PF3D7_0720700**	phosphoinositide-binding protein, putative	S1826
**PF3D7_0802600**	adenylyl cyclase beta	S553, S566
**PF3D7_0815600**	eukaryotic translation initiation factor 3 subunit G, putative	S44
**PF3D7_0821800**	protein transport protein SEC61 subunit beta, putative	S20, S23
**PF3D7_0822900**	conserved *Plasmodium* protein, unknown function	S728
**PF3D7_1008500**	conserved *Plasmodium* membrane protein, unknown function	S355
**PF3D7_1008800**	nucleolar protein 5, putative	S410
**PF3D7_1010300**	succinate dehydrogenase subunit 4, putative	S104
**PF3D7_1011800**	PRE-binding protein	T797
**PF3D7_1012700**	NLI interacting factor-like phosphatase, putative	S1200
**PF3D7_1021700**	conserved *Plasmodium* membrane protein, unknown function	S3231
**PF3D7_1023900**	chromodomain-helicase-DNA-binding protein 1 homolog, putative	T320
**PF3D7_1025900**	conserved *Plasmodium* protein, unknown function	S589
**PF3D7_1026600**	conserved *Plasmodium* protein, unknown function	S174, S324
**PF3D7_1027300**	peroxiredoxin	S217, S226, S228, S229, T230
**PF3D7_1107300**	polyadenylate-binding protein-interacting protein 1, putative	S1430
**PF3D7_1110400**	RNA-binding protein, putative	S435, S1337
**PF3D7_1124600**	ethanolamine kinase	S23, T25
**PF3D7_1125800**	kelch domain–containing protein, putative	S20
**PF3D7_1133400**	apical membrane antigen 1	S610
**PF3D7_1138700**	conserved *Plasmodium* protein, unknown function	S880
**PF3D7_1251200**	coronin	S351
**PF3D7_1343800**	conserved *Plasmodium* protein, unknown function	S6066, S326
**PF3D7_1348600**	conserved *Plasmodium* protein, unknown function	S37
**PF3D7_1413700**	conserved *Plasmodium* protein, unknown function	S1035
**PF3D7_1423300**	serine/threonine protein phosphatase 7	S769
**PF3D7_1455300**	conserved *Plasmodium* protein, unknown function	S14, S18, S136, S292

Abbreviations: *ACβ*, adenylyl cyclase beta; cAMP, cyclic AMP; DHHC2, Asp-His-His-Cys2; FACT, facilitates chromatin transcription; HAD, haloacid dehydrogenase; NLI, nuclear LIM interactor; *PDEβ*, phosphodiesterase beta; *PKA*, cAMP-dependent protein kinase; PRE, Prx regulatory element; ROM4, rhomboid protease 4; SEC61, secretory protein 61; SPT16, suppressor of Ty 16.

Of particular interest, we detected a number of ACβ- and PKA-dependent phosphosites in proteins with known functions in invasion (Fig 7A and 7B). These sites included Ser_610_ within the short cytoplasmic domain of AMA1, which was specifically enriched in the DMSO-treated compared with the RAP-treated samples derived from both the *ACβ-HA*:*loxP* and *PKAc-HA*:*loxP* lines (Fig 7B). This is consistent with previous evidence that phosphorylation at AMA1 Ser_610_ is mediated by PKA [23]. In contrast, although it has previously been suggested that phosphorylation of Ser_19_ of the actinomyosin motor protein myosin A (MyoA) is also PKA mediated [12], we quantified abundant phosphorylated Ser_19_ (pSer_19_) MyoA in both DMSO and RAP-treated *ACβ-HA*:*loxP* and *PKAc-HA*:*loxP* samples (Fig 7B and S2C Fig). This was confirmed by western blot using a phospho-specific antibody against pSer_19_ (S2E Fig). It was concluded that this particular site can be phosphorylated by kinases other than PKA. This is consistent with our previous findings that phosphorylation of MyoA Ser_19_, although PKG dependent, is also calcium dependent, suggesting it may be the substrate of a CDPK [25].

In addition to AMA1 Ser_610_, we identified a number of protein targets of PKA-dependent phosphorylation that have previously been implicated in invasion. One such protein, coronin, has been shown to modulate actin dynamics in *P*. *berghei* sporozoites, with PKA-dependent phosphorylation of this protein being implicated as a mediator of a ‘switch’ from migration to invasion [37]. Coronin also associates with actin in *P*. *falciparum* merozoites [38], so it is possible that PKA-dependent coronin phosphorylation also promotes invasion in asexual blood stages. We also found that phosphorylation of ACβ (Ser_553_ and Ser_566_) and two putative protein phosphatases (PF3D7_1423300 Ser_769_ and PF3D7_1012700 Ser_1200_) are likely cAMP dependent, indicating potential for feedback loops between the enzymes that regulate cAMP-dependent phosphorylation. It is important to note that our subgroup of high-confidence PKA-regulated sites also includes proteins of diverse putative function, including roles in chromatin organisation, RNA binding, translation initiation, ubiquitin metabolism, and protein transport, along with 14 proteins, the functions of which are currently unknown. We also note that most phosphosites in the *Plasmodium* subtilisin-like protease 2 (SUB2) and rhomboid protease 4 (ROM4) proteases were detected at similar levels in ACβ- and PKA-deficient parasites compared with controls (S1 Table). This indicates that the abundance of these enzymes (known to mediate the shedding of proteins from the merozoite surface) is unaffected in our knockouts and cannot account for the reduction in AMA1 shedding that we observe in the absence of PKA activity.

### AMA1 Ser_610_ phosphorylation induces a structural change in the AMA1 cytoplasmic tail

Our data supporting a role for cAMP and PKA in phosphorylation of the AMA1 cytoplasmic domain residue Ser_610_, together with the previous evidence that this modification is important for AMA1 function in erythrocyte invasion [23], led us to further explore the functional consequences of AMA1 Ser_610_ phosphorylation. To do this, we first generated a recombinant form of the AMA1 cytoplasmic domain fused to glutathione S transferase (called GST-AMA1_cyt_). We then assessed the capacity of this protein to be phosphorylated in vitro by mammalian PKA, as well as the effects of any phosphorylation on its structure. As shown in S2F Fig, GST-AMA1_cyt_ was efficiently phosphorylated by murine PKA, and this phosphorylation was dependent on the presence of Ser_610_. Remarkably, further examination of the free recombinant AMA1_cyt_ domain (cleaved from its GST fusion partner) showed that PKA-mediated phosphorylation resulted in a significant conformational change to AMA1_cyt_, detectable by both circular dichroism (CD) (Fig 7C) and nuclear magnetic resonance (NMR) spectroscopy (Fig 7D and Fig 7E). Both methods confirmed that, upon phosphorylation, AMA1_cyt_ undergoes a transition from an unfolded state to a more folded state with an increased helical content. We conclude that phosphorylation of AMA1 Ser_610_ by PKA can induce structural changes that may be involved in signalling functions and/or recruitment of partner proteins important for the function of AMA1 at invasion. Impairment of this process may be partially or wholly responsible for the invasion defect observed upon ablation of ACβ or PKA.

## Discussion

In this study, we have shown that cAMP and PKA are critical components of the signalling cascade(s) required by *P*. *falciparum* merozoites to invade erythrocytes. Parasites deficient in cAMP synthesis or PKA activity are arrested at a late stage of the invasion pathway. In contrast, the known essential steps that precede invasion, including egress, the associated calcium flux, and discharge of merozoite secretory organelles, all occur in the absence of cAMP-dependent signalling.

Our findings have several important implications. First, they temporally separate the essential roles of cGMP-dependent signalling, which triggers merozoite egress [2], from those of cAMP-dependent signalling, which we show here to be critical only for invasion. This conclusion is particularly notable in light of recent evidence from phosphoproteomic, pharmacological, and genetic studies in *T*. *gondii* that suggest interplay between cAMP and cGMP signalling during egress in that parasite. PKAc-deficient tachyzoites were found to egress prematurely and could not stably enter host cells [39,40]. We did not observe similar phenomena in ACβ- or PKAc-null *P*. *falciparum*, implying fundamental differences between these genera in the mechanisms controlling egress and how these are regulated by cyclic nucleotides; whereas cAMP-mediated signalling via *T*. *gondii* PKA appears to negatively regulate egress, this is not the case in *P*. *falciparum*. Indeed, because ACβ- but not PKAc-deficient *P*. *falciparum* parasites displayed a subtle delay in egress, our results in fact imply that cAMP could be a positive regulator of egress through a PKA-independent route, potentially via cross talk with calcium signalling pathways [41]. We suggest that the recently reported dual-specificity phosphodiesterase activity of PDEβ [25] could explain why we see a delay in egress in the absence of cAMP; in wild-type parasites, PDEβ likely contributes to the regulation of levels of both cAMP and cGMP, whilst in the absence of cAMP (in the ACβ-null parasites), there may be increased breakdown of cGMP, leading to delayed and/or inefficient activation of PKG.

Second, our findings lead us to reassess previously proposed mechanisms underlying the essentiality of cAMP-dependent signalling. Earlier studies by others have suggested the involvement of cAMP- and PKA-dependent signalling in early blood stage development in processes such as regulation of ion transport across the host erythrocyte membrane [42] and in the regulation of the cell cycle [41,43]. These studies indicated a complex interplay between cAMP and calcium signalling in *P*. *falciparum* trophozoites. Whilst these developmental processes prior to schizont stage may indeed be fine-tuned through cAMP- and calcium-dependent signalling, we did not observe a critical role for cAMP and PKA in the erythrocytic life cycle until the point of merozoite invasion. Although inhibition of cAMP production has been previously reported to block invasion [16], our study clearly shows that the signalling pathways through which this occurs need to be redefined. This is because, in contrast to the findings of Dawn and colleagues, we have now clearly demonstrated that calcium release and subsequent microneme secretion occur efficiently in the absence of cAMP or PKA, and that PfEpac is not a key mediator of these processes. Because PfEpac also lacks many of the canonical features of EPAC proteins—including the domains required to recruit a Rap1 GTPase central to the proposed mechanism of action [44,45]—we suggest that it is unlikely to be a functional orthologue of mammalian EPAC.

Third, our data provide the first genetic evidence for a mechanistic link between the activity of PKA and the function of AMA1. We correlate the loss of PKA-mediated phosphorylation of AMA1 at Ser_610_ with a reduction in shedding of AMA1, complementing our previous observations that PDEβ-deficient parasites displaying hyper-activation of PKA shed AMA1 prematurely [25]. AMA1 shedding in *P*. *falciparum* is thought to be mediated primarily by the activity of the subtilisin-like protease SUB2, with some contribution from rhomboid proteases [46–48]. Previous attempts to generate parasite mutants from which AMA1 cannot be shed have been unsuccessful, suggesting that shedding is important [49]. It is possible that a finely tuned amount of merozoite surface AMA1 is required for efficient invasion; too large a quantity of AMA1 on the parasite surface may impede the binding of RON2 or conversely lead to too many AMA1-RON2 interactions to allow the tight junction to move efficiently around the invading merozoite, whereas a smaller amount may be sufficient to establish a tight junction within which the AMA1 component could then be protected from cleavage in a manner analogous to that described in *T*. *gondii* [50]. In this scenario, timely activation of PKA may be critical so as to ensure the optimal amount of surface AMA1 at the point at which the merozoite makes contact with the erythrocyte. Our demonstration that PKA-dependent phosphorylation of Ser_610_ results in a dramatic conformational transformation of the AMA1 cytoplasmic tail such that it adopts a more folded structure tempts us to speculate that this structural transition could promote interactions with, and/or the activation of, the enzymes that mediate AMA1 shedding. This may not be required absolutely for shedding but could modulate the rate of this process.

The relationship between the deficiency in AMA1 shedding and the block in invasion we observe in cAMP- and PKA-deficient parasites remains to be determined. Our findings suggest that the mutant parasites secrete adhesins from micronemes and rhoptries, and deform host RBCs via the activity of the actinomyosin invasion motor. However, the final stages of entry into the host cell downstream of tight junction formation are inhibited. A similar late-stage block in invasion has been observed when merozoites are released in the presence of small peptides that bind to AMA1 and block the interaction with RON2 [24,51,52], suggesting that the block in invasion we observe in the absence of cAMP-dependent signalling might at least in part be explained by a direct effect on AMA1 function. However, our phosphoproteome analyses demonstrate cAMP- and PKA-dependent phosphorylation of a large number of other parasite proteins, some of which have previously been suggested to play a role in invasion. We therefore suggest that there are likely to be multiple mechanisms by which cAMP-dependent signalling controls invasion, including but not necessarily limited to modulation of AMA1 shedding and function.

Whilst the full range of biological functions of specific cAMP- and PKA-dependent phosphorylation events remain to be addressed, our findings demonstrate the fundamental importance of the cAMP signalling pathway in *P*. *falciparum* asexual blood stages and validate ACβ, PKA, and PDEβ as candidate targets for new approaches to antimalarial drug discovery.

## Methods

### *P*. *falciparum* culture and synchronisation

*P*. *falciparum* erythrocytic stages were cultured in human erythrocytes (National Blood Transfusion Service, UK) and RPMI 1640 medium (Life Technologies) supplemented with 0.5% Albumax type II (Gibco), 50 μM hypoxanthine, and 2 mM L-glutamine. Synchronous parasite cultures were obtained as described previously [46]. Briefly, segmented schizonts were enriched by centrifugation on a 70% Percoll (GE Healthcare) cushion, followed by the addition of fresh erythrocytes to allow invasion for 1–2 h under continuously shaking conditions. Residual intact schizonts were then removed by a further cycle of Percoll treatment and the resulting pellet treated with sorbitol to yield highly synchronous ring-stage cultures. In all cases, induction of DiCre activity when required was by treatment for 2–4 h with 100 nM RAP (Sigma) as described previously [25,26,33]. Control parasites were treated with vehicle only (1% v/v DMSO).

### Genetic modification of *P*. *falciparum* parasites

The *ACβ-HA*:*loxP* parasite line was generated by Cas9-mediated genome editing of the DiCre-expressing B11 *P*. *falciparum* clone, as described previously [33]. A C-terminal triple-HA tag and loxP site were added to the *ACβ* gene using a repair template containing a 5′ homology arm containing 840 bp of sequence from the 3′ end of *ACβ* exon 3, a 480-bp re-codonised region corresponding to the sequences of exons 4 and 5, triple-HA and loxP sequences, and an 846-bp 3′ homology arm derived from the *ACβ* 3′UTR. This repair template was synthesised commercially (Geneart; Thermo), linearised immediately upstream of the 5′ homology region by digestion with SpeI, and transfected in conjunction with a pDC2-based plasmid [53] encoding Cas9 and a single guide RNA (sgRNA) targeted to ATTGCATGTCCCTAATCGAT at the 5′ end of the fourth exon. Clones expressing HA-tagged ACβ were isolated by limiting dilution and subsequently transfected to replace the second intron of the modified *ACβ* gene with a loxP-containing *SERA2* intron (*SERA2loxPint*) [54], again using the Cas9 system. The repair construct for this modification step comprised the *SERA2loxPint* module followed by 300 bp of re-codonised sequence corresponding to the 5′ end of exon 3 and was flanked by approximately 500 bp homology regions. This repair template was linearised in the same manner using a SpeI site upstream of the 5′ homology region. The corresponding sgRNA was targeted to GAGACGCCGTTCTTGTTATA at the 5′ end of exon 3. Doubly modified clones were obtained by limiting dilution and confirmed by diagnostic PCR and capillary sequencing.

The *PKAc-HA*:*loxP* line was generated from the DiCre-expressing 3D7 [53] *P*. *falciparum* clone using SLI of a plasmid based on pL7 (a kind gift from Kathrin Witmer, Imperial College London), in which the yFCU expression cassette of pL6 [55] had been deleted. The gRNA cassette from pL7 was removed and replaced with a synthetic cassette containing a *SERA2loxPint* followed by a triple-HA tag and downstream loxP, glucosamine-6-phosphate riboswitch ribozyme (glmS), and *PbDT* 3′UTR sequences (IDT). A fragment containing a thosea asigna virus 2A peptide (T2A) ribosomal skip peptide and NeoR cassette was amplified from a pSLI-sandwich plasmid [28] and cloned downstream of the C-terminal triple HA. A re-codonised version of PKAc exons 4 and 5 was synthesised commercially (IDT) and inserted downstream of the *SERA2loxPint* and upstream of the 3×HA tag. An 800-bp 5′ homology region comprising exon 2, intron 2, and exon 3 of the endogenous PKAc locus was cloned upstream of the *SERA2loxPint*. Following transfection of purified schizonts using an AMAXA nucleofector 4D (Lonza) and P3 reagent, modified parasites were selected as described previously [28] and cloned by limiting dilution.

The *PKAc-HA*:*loxP* line was further modified to produce the complemented *PKAc-HA_DDDcomp*:*loxP* line. A plasmid based on a custom DiCre-inducible expression vector (pDCIn) was used to integrate sequence encoding a RAP-inducible, triple-HA tagged PKAc fused to a DDD into the *p230p* locus using Cas9-mediated gene editing with a gRNA previously reported for this locus [53]. pDCIn was generated by modifying pBCam by several cloning steps using the NEBuilder Gibson assembly. First, an eGFP gene was amplified with a T2A peptide in the frame, with the C terminus and a MluI restriction site followed by loxP site preceding the eGFP coding sequence. This was cloned in frame with the *BSD* gene from pBCam using a BstBI site. The Cam promoter was excised using PstI and SalI sites and a second loxP site preceded by a multiple cloning site inserted upstream of the Hrp2 3′UTR. The 800-bp homology regions for the *p230p* locus were inserted using a SmaI site for the 5′ homology region and an EcoRI site for the 3′ homology region. This yielded pDCIn (DiCre induction). To modify this to suit PKAc complementation, a 2-kbp region of the *PKAc* 5′UTR was cloned into the plasmid upstream of the GFP T2A BSD cassette using MluI and SmaI sites. Finally, a synthetic DNA fragment consisting of a re-codonised *PKAc* coding sequence followed by triple-HA and DDD sequences (IDT) was inserted downstream of an EGFP T2A BSD^R^ expression cassette (driven by the cloned PKAc 5′UTR) and a second loxP site using NotI and KpnI sites. This plasmid (10 μg) was linearised by digestion with AatII close to the ampR cassette and co-transfected together with the pDC2p230p Cas9/sgRNA-containing plasmid (50 μg) into *PKAc-HA*:*loxP* parasites, as previously described [53]. The transfected culture was treated with 5 μg/mL BSD (Sigma) (3 d post-transfection to select for integration. EGFP-positive parasites, indicative of successful integration of the construct, were observed by live microscopy (S1C Fig), and BSD selection was continued until no WT *p230p* locus could be detected by PCR. Upon treatment with RAP, parasites were expected to switch from expressing EGFP and BSD from the *p230p* locus and PKAc-HA_3_ from the *PKAc* locus to expressing PKAc-HA_3_-DDD from the *p230p* locus and a truncated, untagged N-terminal fragment of PKAc from the *PKAc* locus. These transitions were verified by live fluorescence microscopy and western blotting, which showed a switch in molecular mass of the HA-positive band from approximately 50 kDa (the mass of PKAc-HA_3_ expressed from the *PKAc* locus) to approximately 61 kDa (the approximate mass for PKAc-HA_3_-DDD) in the presence of 10 μM TMP, which is required to stabilise PKAc-HA_3_-DDD. The PfEPAC knockout plasmid was constructed by cloning the first 800-bp homology region *EPAC* coding sequence into the pSLI DiCre plasmid in frame with the downstream T2A NeoR cassette (Fig 4D). Following transfection of purified schizonts using an AMAXA nucleofector 4D (Lonza) and P3 reagent, modified parasites were selected as described previously [28], applying G418 selection until no WT parasites were detected by PCR. All plasmid sequences were verified by capillary sequencing, and all RAP treatments were performed on ring-stage parasites.

Oligonucleotide primers used in diagnostic PCR to detect integration and excision of transgenes, and the sequences of re-codonised regions, are provided below in Tables 2 and 3.

**Table 2:** Sequences of PCR primers.

**ACβ primers (Fig 1A and 1B)**	**Name**	**Sequence**
**PCR1 Forward**	SERA2loxPint_F (AJP_166)	GCATACATTATACGAAGTTATTATATATG
**PCR1 Reverse**	ACβ_exon3_R (AJP_244)	GCATGTCCCTTGAACCATAACTTTGTC
**PCR2 Forward**	ACβ_exon3_F (AJP_051)	GAACAGACCAATCAACAGAAC
**PCR2 Reverse**	HA_R	GGCATAGTCCGGGACGTC
**PCR3 Forward**	ACβ_exon2_F (AJP_135)	AGCAAATGTGAAAACCCGGCACAG
**PCR3 Reverse**	ACβ_3′UTR_R (AJP_103)	CGAGTAGGGAGCATAACAAATAG
**PKAc primers (Fig 2A and 2B)**	**Name**	**Sequence**
**PCR1 Forward**	PKAc 5′ Int F	GAAGGACAGTGATTCTAGTGAACAG
**PCR1 Reverse**	PKAc WT R	CAATTTCTTCATCAAATGTTTGCAATTGTTATC
**PCR2 Forward**	PKAc 5′ Int F	GAAGGACAGTGATTCTAGTGAACAG
**PCR2 Reverse**	PKAc 5′ Int R	GTTCTGTGCACCCTTCTTAAGG
**PCR3 Forward**	PKAc 3′ Int F	CAGCTATGACCATGATTACGCC
**PCR3 Reverse**	PKAc 3′ Int R	GTTAAGTATTACCTTAATAAAAATATTGTATG
**PCR4 Forward**	PKAc excision F	GAATGAAAATGTTCAGGTTCCTTTG
**PCR4 Reverse**	PKAc excision R	CCGTTCAAATCTTCTTCAGAAATCAAC
**PKAc complementation primers (Fig 3C and S1D Fig)**	**Name**	**Sequence**
**P230p locus Forward**	P230p Int F	CTATATGGTATCCAAAACCTTTAAATTATATAGC
**P230p locus Reverse**	P230p WT R	GAGGAATTTTTAAATATGATATACCTTTATCATTAG
**PCR1 Forward**	P230p Int F	CTATATGGTATCCAAAACCTTTAAATTATATAGC
**PCR1 Reverse**	P230p 5′ Int R	CTAAATTAGAAAATGAACATATAGAAAGCATC
**PCR2 Forward**	P230p excision F	CACCTTTATGATTTGTTCTGTTACATG
**PCR2 Reverse**	P230p excision R	CGACGCAAAAAGGTGAAAAACTC
**PCR3 Forward**	Hrp2 F	CTTTATGTCGACTCATCTAGGGAAG
**PCR3 Reverse**	P230p 3′ Int R	CATGTGATTTAGTATTAATAACTTTAACTTGATC
**Epac primers (Figs 4D and S1E)**	**Name**	**Sequence**
**PCR1 Forward**	Epac 5′ Int F	GATTAATTCAGAGCAATATAAAAAAGAGAAAAG
**PCR1 Reverse**	Epac 5′ Int R	GCATAGTCAGGAACATCGTAAGG
**PCR2 Forward**	Epac 5′ Int F	GATTAATTCAGAGCAATATAAAAAAGAGAAAAG
**PCR2 Reverse**	Epac WT R	GTTGTTATTTATATTTTCATGAATAGGAC
**PCR3 Forward**	PKAc 3′ Int F	CAGCTATGACCATGATTACGCC
**PCR3 Reverse**	Epac 3′ Int R	GTTGTTATTTATATTTTCATGAATAGGAC

Abbreviations: F, forward; Int, integration; R, reverse; WT, wild-type.

**Table 3:** Sequences of re-codonised regions.

Region	Sequence
***ACβ* re-codonised amino acids F**_**116**_**–N**_**215**_	TTCTTCTGCGATGCTAGTGGCTTTAGCAATCTGGCCGAACAATTAGACAAACGTATCAATGGGACCGAACTCCTCGGAAATTGTCTCAACAAGTTCTTCAACATCCTGATCAAAATCATCGACTATTGGGGGGGCGATATTATTAAGTTTAGTGGTGATGCTGTATTGGTGATCTGGCCGTTGCACAACGTATTAAAAAACAAAAAGAAGAAAAAGAAAAAAAACGGGACCCAAAATAACGACAGTGATGATGAGCATGTCAACTTGAAGGACAACAAAACCTCGCACGATCGTTATAAT
***ACβ* re-codonised amino acids T**_**2,125**_**–S**_**2,279**_	ACAGCATACTGTATGAGTTTGATTGACAGTCTGAATCAGGAGGAGCAATTACTCGCGAAATTATGTTCATTCTTCAACAATACGTTCAACATTAAGAAAATGGAGTGCATTTACCCGAAGTACATCTCTCGCTGTGAATTGAAGAAAATCATTGTTAAACTGGTGGAGAAAAATGTATTTTGTTTGTACGAGGATCCTAACAAAAAGTCTGTGACCCTTCCTAAGGACAATGTTAACAGCATCCACAACATTAATTTCGTCTATCGCGACATGTATAATAAGATCAATAGCTTTTTGGAGAAAAAATCCAAACATCATTCACTTTTCGGAAAGCATAAGAACGTCCCGGACGAAGAGATTTACTTTTGCATCACAAATTTTTCCTTGAAGAAAGTCTTGAACGACCTGCTTGAAAACGAGGAGAAAGAGTACATCAAAAAGATCTATAAGAAGTACATTGAATCC
***PKAc* re-codonised amino acids A**_**211**_**–W**_**342**_	GCAGACTGGTGGACACTTGGAATATTTATTTATGAGATTCTTGTAGGTTGCCCTCCATTCTACGCTAACGAGCCTCTTCTTATATACCAAAAGATTCTTGAGGGAATTATTTACTTTCCAAAGTTCCTAGACAATAACTGTAAGCACCTAATGAAAAAGCTACTAAGTCATGACCTTACTAAGCGTTACGGAAACCTTAAGAAGGGTGCACAGAACGTAAAGGAGCATCCTTGGTTCTCTAACATAGACTGGGTTAACCTACTTAACAAGAACGTAGAAGTACCTTACAAGCCAAAGTACAAGAACATATTCGACTCTTCTAACTTCGAACGTGTTCAGGAGGACCTTACAATTGCAGACAAGATTACTAACGAGAACGACCCTTTCTACGACTGG

### Parasite sample preparation and western blot

Parasite culture supernatant samples for egress and adhesin shedding assays were prepared from tightly synchronised cultures. Percoll-enriched mature schizonts were resuspended in complete medium containing the PKG inhibitor 4-[7-[(dimethylamino)methyl]-2-(4-fluorphenyl)imidazo[1,2-α]pyridine-3-yl]pyrimidin-2-amine (compound 2 or C2; 1.5 μM) and allowed to further mature for 3 h until predominantly mature segmented schizonts. Schizonts were then pelleted by centrifugation at 800*g*, washed to remove the PKG inhibitor, and suspended at a 10% haematocrit in fresh warm medium. Aliquots (100 μL) were harvested at specified time points; schizonts were pelleted by centrifugation and culture supernatants collected and clarified using 0.22-μm Costar Spin-X centrifuge filters (Corning). The schizont pellet from *t* = 0 was retained as a pellet control sample.

Parasite extracts were prepared from Percoll-purified schizonts treated with 0.15% w/v saponin to remove erythrocyte material. To solubilise parasite proteins, washed saponin-treated parasite pellets were resuspended in three volumes of NP-40 extraction buffer (10 mM Tris, 150 mM NaCl, 0.5 mM EDTA, 0.5% NP40, pH 7.5, with 1× protease inhibitors (Roche). Suspensions were incubated on ice for 10 min followed by centrifugation at 12,000*g* for 10 min at 4°C. For western blot, SDS-solubilised proteins were electrophoresed on 4%–15% Mini-PROTEAN TGX Stain-Free Protein Gels (Bio-Rad) under reducing conditions and proteins transferred to nitrocellulose membranes using a semidry Trans-Blot Turbo Transfer System (Bio-Rad). Antibody reactions were carried out in 1% skimmed milk in PBS with 0.1% Tween-20 and washed in PBS with 0.1% Tween-20. Appropriate horseradish peroxide-conjugated secondary antibodies were used, and antibody-bound washed membranes were incubated with Clarity Western ECL substrate (Bio-Rad) and exposed to X-ray film for visualisation.

Antibodies used for western blots presented in this work were as follows: anti-HA monoclonal antibody (mAb) 3F10 (diluted 1:1,000) (Roche); rat anti–binding immunoglobulin protein (BiP) (1:2,000); mouse anti-GAPDH mAb (1:20,000); rabbit anti-SERA5 polyclonal antibody (1:2,000) [56]; rabbit anti-AMA1 (1:2,000) [57]; rabbit anti-EBA175 (1:5,000) [58]; rabbit anti-Rh2b (1:2,000) [59]; mouse anti-MSP1_83_ mAb (1:5,000) [60]; mouse anti-plasmepsin V mAb (1:2,000); rat anti-myosin A serum (1:10,000) [3]; and rabbit anti-pS_19_MyoA antibodies (1:1,000) [25]. Densitometry quantifications were performed using Image J.

### IFAs

Thin blood films were fixed with 4% formaldehyde in PBS and permeabilised with PBS containing 0.1% (v/v) Triton X-100. Blocking and antibody binding was performed in PBS 3% BSA w/v at room temperature. Slides were mounted with ProLong Gold Antifade Mountant containing DAPI (Thermo Fisher Scientific). Images were acquired with a NIKON Eclipse Ti fluorescence microscope fitted with a Hamamatsu C11440 digital camera and overlaid in ICY bioimage analysis software or Image J. Super-resolution images were acquired using a Zeiss LSM880 confocal microscope with Airyscan detector in Airyscan SR mode. Antibodies additionally used for IFA not described above were rabbit anti-ARO and rabbit anti-GAP45 polyclonal antisera (both diluted 1:1,000), and a mouse anti-RON4 polyclonal antiserum (1:500) [19]. To visualise tight junction formation, mature schizonts of DMSO- and RAP-treated *PKAc-HA*:*loxP* parasites were Percoll enriched, incubated in medium containing C2 (1.5 μM) for 2 h, and then washed in warm medium and further incubated in the presence of fresh erythrocytes at 5% haematocrit for 30 min. In the case of the DMSO-treated control parasites, the medium contained 1 μM cytochalasin D to prevent invasion but allow junction formation. The cultures were then rapidly fixed by adding an equal volume of PBS containing 8% formaldehyde and 0.02% glutaraldehyde and shaking at 37°C for 20 min. Fixed parasites were then processed, as previously described, using the rabbit anti-AMA1 primary antibody at a dilution of 1:500.

### Time-lapse video microscopy

Egress and invasion were monitored by differential interference contrast (DIC) microscopy using a Nikon Eclipse Ni light microscope fitted with a Hamamatsu C11440 digital camera. Egress videos were performed using one population of parasites stained briefly with Hoechst, as described previously [33]. Invasion videos were performed using schizonts purified from DMSO- or RAP-treated *ACβ-HA*:*loxP* or *PKA-HA*:*loxP* cultures mixed with uninfected erythrocytes. DIC images were taken every 150 ms for at least 8 min, and the resulting time-lapse videos were processed using Nikon NIS Elements AR analysis software.

### Flow cytometry

For growth assays, synchronous ring-stage parasites were adjusted to a 0.1% parasitaemia 1% haematocrit suspension and dispensed in triplicate into six-well plates. Samples of 100 μL were harvested at days 0, 2, and 4 for each well and fixed with 4% formaldehyde 0.2% glutaraldehyde in PBS. Fixed samples were stained with SYBR green and analysed by flow cytometry.

### TEM

To enrich for attachment of PKAc-null merozoites to the surface of erythrocytes, RAP-treated PKAc-HA:loxP cultures were allowed to mature to schizont stage and then added to an excess of fresh erythrocytes and shaken gently at 37°C for 40 min. The cultures were then pelleted and resuspended in fixative (2% formaldehyde, 1% glutaraldehyde in PBS, pH 7.4) at 37°C for 15 min. Fixed material was briefly washed in PBS before mixing with 20% (w/v) dextran in complete medium containing bakers’ yeast, then freezing using a HPM100 high-pressure freezer (Leica). Vitrified cells were freeze-substituted using an EM AFS2 (Leica) into Lowicryl HM20 resin (EMS) with 0.2% (w/v) uranyl acetate and cut into 120-nm sections using a UC7 microtome (Leica). Sections were placed on glow-discharged carbon-coated copper finder grids (EMS) and post-stained with 0.2% (w/v) uranyl acetate and 4% (w/v) lead citrate. Images were recorded with a Tecnai T12 120-kV field emission gun electron microscope (FEI) equipped with a 4k × 4k Ultrascan 4000 CCD camera (Gatan). Tomograms were recorded using a Model 2040 dual-axis tomography holder (Fischione Instruments). Dual-axis tilt series were acquired from −60° to +60° with an increment of 2° using SerialEM [61]. Tomograms were processed in IMOD [62] using patch tracking for image alignment, and the final reconstruction was filtered using nonlinear anisotropic diffusion filtering.

### Phosphoproteomics

The phosphoproteomics data presented are from two isobaric labelling experiments, the first involving *ACβ-HA*:*loxP*–derived samples and the second using *PKAc-HA*:*loxP*. Tightly synchronised, ring-stage *ACβ-HA*:*loxP* or *PKAc-HA*:*loxP* parasites were treated with 100 nM RAP or vehicle only (DMSO) and schizonts (about 45 h old) enriched on an approximately 70% Percoll cushion. The schizonts were treated for 2 h with 1 μM C2 (to arrest egress) and then washed to allow egress for 30 min, after which the cultures were treated with 0.15% saponin in PBS containing cOmplete Mini EDTA-free Protease and PhosSTOP Phosphatase inhibitor cocktails (both Roche) for 10 min at 4°C to lyse the host erythrocytes. Samples were washed twice in PBS containing protease and phosphatase inhibitors, snap-frozen in liquid nitrogen, and pellets stored at −80°C. Parasite pellets were resuspended in 1 mL 8 M urea in 50 mM HEPES, pH 8.5, containing protease and phosphatase inhibitors and 100 U/mL benzonase (Sigma). Proteins were extracted from the pellets using three 15-s bursts with a probe sonicator followed by a 10-min incubation on ice and a 30-min centrifugation at 14,000 rpm at 4°C. Protein content was estimated by a BCA protein assay and a 200-μg aliquot of each sample was taken for further processing. Samples were reduced with 10 mM dithiothreitol for 25 min at 56°C and then alkylated with 20 mM iodoacetamide for 30 min at room temperature. The alkylation reaction was quenched with an additional 10 mM dithiothreitol, and then each sample was diluted with 50 mM HEPES to reduce the urea concentration to <2 M prior to digestion. Proteolytic digestion was carried out by the addition of 4 μg LysC (WAKO) and incubated at 37°C for 2.5 h followed by the addition of 10 μg trypsin (Pierce) and overnight incubation at 37°C. After acidification, C_18_ MacroSpin columns (Nest Group) were used to clean up the digested peptide solutions and the eluted peptides dried by vacuum centrifugation. Samples were resuspended in 50 mM HEPES and labelled using the 0.8 mg Tandem Mass Tag 10plex isobaric reagent kit (Thermo Scientific) resuspended in acetonitrile. Labelling reactions were quenched with hydroxylamine, and a pool was made of each set of samples. Acetonitrile content was removed from the pooled TMT solution by vacuum centrifugation and then acidified before using a Sep-Pak C_18_ (Waters) to clean up the labelled peptide pool prior to phosphopeptide enrichment. The eluted TMT-labelled peptides were dried by vacuum centrifugation and phosphopeptide enrichment was subsequently carried out using the sequential metal oxide affinity chromatography (SMOAC) strategy with High Select TiO_2_ and Fe-NTA enrichment kits (Thermo Scientific). Eluates were combined prior to fractionation with the Pierce High pH Reversed-Phase Peptide Fractionation kit (Thermo Scientific). The dried TMT-labelled phosphopeptide fractions generated were resuspended in 0.1% TFA for LC-MS/MS analysis using a U3000 RSLCnano system (Thermo Scientific) interfaced with an Orbitrap Fusion Lumos (Thermo Scientific). Each peptide fraction was pre-concentrated on an Acclaim PepMap 100 trapping column before separation on a 50-cm, 75-μm I.D. EASY-Spray Pepmap column over a 3-h gradient run at 40°C, eluted directly into the mass spectrometer. The instrument was run in data-dependent acquisition mode with the most abundant peptides selected for MS/MS fragmentation. Two replicate injections were made for each fraction with different fragmentation methods based on the MS^2^ HCD and MSA SPS MS^3^ strategies described [63]. The acquired raw mass spectrometric data were processed in MaxQuant [64] (version 1.6.2.10) for peptide and protein identification; the database search was performed using the Andromeda search engine against the *Homo sapiens* canonical sequences from UniProtKB (release 2018_05) and *P*. *falciparum* 3D7 sequences from PlasmoDB-39. Fixed modifications were set as Carbamidomethyl (C) and variable modifications set as Oxidation (M) and Phospho (STY). The estimated false discovery rate was set to 1% at the peptide, protein, and site levels. A maximum of two missed cleavages were allowed. Reporter ion MS^2^ or Reporter ion MS^3^ was appropriately selected for each raw file. Other parameters were used as preset in the software. The MaxQuant output file PhosphoSTY Sites.txt, an FDR-controlled site-based table compiled by MaxQuant from the relevant information about the identified peptides, was imported into Perseus (v1.4.0.2) for data evaluation. Sites described as ‘enriched’ in the text are those that were quantified more highly in the three DMSO-treated samples compared with the three RAP-treated and *p* < 0.05 (Welch *t* test, two sided, S0 = 0). Sites described as significantly changed in the text are those that were quantified more highly in the three DMSO-treated samples compared with the three RAP-treated, *p* < 0.05 (Welch *t* test, two sided), and are still considered changed when S0 is set to 0.2 in Perseus. Sequence logos were generated using IceLogo (https://iomics.ugent.be/icelogoserver/). The mass spectrometry proteomics data have been deposited to the ProteomeXchange Consortium (http://proteomecentral.proteomexchange.org) via the PRIDE partner repository [65] with the dataset identifier PXD012143.

### Recombinant AMA1_cyt_ protein expression, preparation, and phosphorylation

Recombinant AMA1 cytoplasmic tail protein (residues 567–622) with a thrombin-cleavable GST fusion (GST-AMA1_cyt_), or an AMA1 Ser_610_Ala point mutant of the same protein (GST-AMA1_cyt_ _S610A), was expressed in *Escherichia coli* BL21 and purified as described previously [23]. For NMR experiments, labelled protein was produced using *E*. *coli* grown in M9 medium containing ^15^N ammonium sulphate and ^13^C glucose as the sole nitrogen and carbon sources. To cleave the GST component, 100 μg GST-AMA1_cyt_ protein was treated with one unit of human alpha-thrombin (HTI) overnight at 18°C in 50 mM Tris-HCl, pH 8.2, and 2 mM CaCl_2_. Following the digestion, glutathione agarose (Sigma) was used in excess to trap GST in solution and was later removed by centrifugation. The protein solution was then passed through a Superdex 75 HR 26/60 column equilibrated with 20 mM Tris-HCl, pH 8.2, and 150 mM NaCl to remove thrombin and residual GST. For phosphorylation, 200 μg of AMA1_cyt_ in 20 mM Tris-HCl, pH 8.2, and 150 mM NaCl was treated with 3.5 μg of mouse PKAc-α (Bioaffin GmbH & Co KG) in the presence of 2 mM ATP, 20 mM MgCl2, and 2 mM DTT and incubated overnight at 30°C. The protein solution was then passed through a Superdex 75 HR 26/60 column equilibrated in 20 mM Tris-HCl, pH 8.2, and 150 mM NaCl. This step completely removed PKAc-α.

### CD

Far-UV CD spectra were recorded on a Jasco J-815 spectropolarimeter fitted with a CDF-426S Peltier unit. CD measurements of all GST fusion proteins (free GST, GST-AMA1_cyt_, and GST-AMA1_cyt__pS_610_) were made at a protein concentration of 0.15 mg/mL in 20 mM Tris-HCl, pH 8.2, 150 mM NaCl, using fused silica cuvettes with 1-mm path lengths (Hellma). Spectra were typically recorded with 0.1-nm resolution, with the baseline corrected by subtraction of the appropriate buffer spectrum and the contribution of GST subtracted from the unphosphorylated and PKA-phosphorylated GST-AMA1_cyt_ protein fusions for each secondary structure element (alpha, beta, turn, and random) at the residue level. CD intensities are presented as the CD absorption coefficient calculated on a mean residue weight basis (Δε_MRW_). Secondary structure content was estimated using methods described previously [66].

### NMR spectroscopy

NMR experiments were performed on uniformly ^15^N- and ^13^C-labelled samples at 25°C in 50 mM Tris, 150 mM NaCl on Bruker 600-, 700-, and 800-MHz spectrometers equipped with pulsed-field gradient units and triple resonance probes. Chemical shifts (^1^H, ^15^N, and ^13^C) and NOEs of AMA1_cyt_ and AMA1_cyt__pSer_610_ were determined by performing standard triple resonance experiments [67]. NMR data were processed with NMRPipe/NMRDraw [68] and analysed with XEASY [69]. TALOS+ [70] was used to determine the secondary structure propensity of pAMA-1 on the basis of the measured ^1^H, ^15^N, ^13^Cα, ^13^Cβ, and ^13^CO chemical shifts.

### Author contributions

Unless otherwise stated all experiments were designed and carried out by AP and/or AJP. The order of co-first authors was determined by coin toss. MJB and DAB supervised the work overall and CF provided intellectual input into the design and interpretation of experiments. HF and AS were involved with the design, execution and analysis of the phosphoproteomic mass spectrometry; CB performed the TEM; MT, TWG, and CWM produced the AMA1cyt recombinant proteins; CWM performed the CD under the supervision of SRM; GN performed the NMR experiments under the supervision of AR.

## Supporting information

S1 Fig (A) Western blots from a subcellular fractionation experiment showing that PKAc-HA_3_ localises predominantly in the soluble fraction of a hypotonic freeze-thaw lysate of *PKAc-HA*:*loxP* schizonts. GAPDH was used as a positive control for the soluble fraction, and plasmepsin V (PMV) was used as a positive control for the integral membrane fraction, which was extracted with SDS/Triton X-144. The peripheral membrane fraction was extracted with 100 mM sodium bicarbonate. (B) Growth curves showing changes in parasitaemia of the parental *3D7 DiCre* line and *PKAc-HA*:*loxP* parasites treated with DMSO (vehicle-only control) or RAP. Means from three replicates are plotted. Error bars, SD. (C) Fluorescence microscopy showing expression of EGFP in *PKAc-HA_DDDcomp*:*loxP* parasites, and subsequent loss of signal following RAP treatment, which switches expression of the protein(s) at this locus from EGFP expression to expression of PKAc-HA_3__DDD. Scale bar, 50 μm. (D) Diagnostic PCR analysis verifying successful modification of the *p230p* locus of the *PKAc-HA*:*loxP* line to generate the *PKAc-HA_DDDcomp*:*loxP* line, and successful excision at the modified *p230p* and *PKAc* loci following treatment with RAP. Priming sites are indicated in Fig 3C, and the PCR used to amplify the *PKAc* locus corresponds to PCR 4 in Fig 2B. (E) Diagnostic PCR verifying successful integration of the transgene used to create the Epac knockout line. Priming sites are indicated in Fig 4D. (F) Western blot verifying expression of an approximately 42-kDa HA_3_-tagged fusion of the extreme N terminus of Epac upon deletion of the rest of the gene by the genetic modification shown in Fig 4D. GAPDH expression is shown as a loading control. (G) IFA verifying expression of an HA_3_ tag fused to the extreme N terminus of Epac upon deletion of the rest of the gene by the genetic modification shown in Fig 4D. Scale bar, 50 μm. (H) Growth curve showing unimpaired proliferation of *PfEpac* knockout parasites. Means from three replicates are plotted. Error bars, SD. Data associated with this figure can be found in the supplemental data file (S1 Data). EGFP, enhanced green fluorescent protein; Epac, exchange protein directly activated by cAMP; GAPDH, glyceraldehyde 3-phosphate dehydrogenase; HA_3_, triple hemagglutinin; IFA, immunofluorescence assay; PKAc, catalytic subunit of cAMP-dependent protein kinase; PMV, plasmepsin V; RAP, rapamycin.
(TIF)

S2 Fig (A) Flow cytometry–based invasion assays showing the progression of DMSO-treated *ACβ-HA*:*loxP* and *PKAc-HA*:*loxP* parasites from schizonts (*t* = 0) through rings (*t* = 2) to trophozoites (*t* = 20), and the lack of progression of the RAP-treated counterparts through these stages. (B) IFA showing re-localisation of AMA1 from micronemes to the merozoite periphery in DMSO- and RAP-treated *ACβ-HA*:*loxP* schizonts. IFA analysis was performed on highly synchronous cultures, which were treated with 20 μM E64 about 44 h post invasion for approximately 4 h. Scale bars, 5 μm. (C) Ratio-intensity plots showing log_10_-transformed signal intensities plotted against the log_2_-transformed fold change in intensity (DMSO/RAP) for each site in the *ACβ-HA*:*loxP* and *PKAc-HA*:*loxP* phosphoproteomic profiling experiments. Sites that conform to a minimal PKA consensus motif (R/K, x, pS/pT) are indicated in red. (D) Motif analysis performed using IceLogo of the 533 31–amino acid regions surrounding phosphosites specifically enriched (Welch *t* test, *p* < 0.05) in DMSO-treated *ACβ-HA*:*loxP−* and *PKAc-HA*:*loxP* parasites compared with their RAP-treated counterparts. All 25,344 phosphosites detected in any sample were used as a reference dataset. Characters below the position line indicate amino acid residues that are unfavoured for those positions. (E) Western blot showing the presence of phosphorylated MyoA Ser_19_ in the absence of PKAc in the *PKAc-HA*:*loxP* line. (F) Coomassie stained gel showing changed mobility of GST-AMA1_cyt_ following treatment with mouse PKA. This shift was not observed in the GST-AMA1_cyt_ _S_610_A mutant. AMA1, apical membrane antigen 1; AMA1_cyt_, AMA1 cytosolic domain; E64, cysteine protease inhibitor; GST, glutathione S transferase; IFA, immunofluorescence assay; MyoA, myosin A; PKA, cAMP-dependent protein kinase; PKAc, catalytic subunit of cAMP-dependent protein kinase; RAP, rapamycin.
(TIF)

S3 Fig (A) Super-resolution immunofluorescence imaging of *PKAc-HA*:*loxP* merozoites attached to the RBC surface. Four merozoites for each condition (DMSO- or RAP-treated) are shown. Scale bars, 2 μm. RAP, rapamycin; RBC, red blood cell.
(TIF)

S4 Fig (A) Quantification of egress of DMSO- and RAP-treated *PKAc-HA*:*loxP* and *ACβ-HA*:*loxP* schizonts, based on densitometry measurements of the SERA5 p50 bands on the blots represented in Fig 4A. Signals are normalised such that the mean signal for the 60-min time point of each DMSO control is equal to one. Means from two replicates are plotted. Error bars, SD. (B) Full-length blots used to compile Fig 6A and Fig 6B. (C) Quantification of AMA1, EBA175, and Rh2b shedding from DMSO- and RAP-treated *PKAc-HA*:*loxP* merozoites, based on densitometry measurements on blots of the type represented in Fig 6A. Signals are normalised such that the mean signal for the 60-min time point of each DMSO control is equal to one. Means from three replicates are plotted. Error bars, SD. (D) Quantification of protein detection in the supernatants of rupturing DMSO- and RAP-treated *PKAc-HA*:*loxP* schizonts from blots of the type shown in Fig 6A. Densitometry measurements are normalised such that the mean signal for each DMSO control is equal to one. Means from three replicates are plotted. Error bars, SD. Data associated with this figure can be found in the supplemental data file (S1 Data). AMA1, apical membrane antigen 1; EBA175, erythrocyte binding antigen 175; p50, processed 50 kDa form; RAP, rapamycin; Rh2b, reticulocyte binding protein homologue 2b; SERA5, serine repeat antigen 5.
(TIF)

S1 Table Phosphoproteomic mass spectrometry datasets. (XLSX)

S1 Data Data values associated with figure plots. (XLSX)

S1 Movie Time-lapse video microscopy of RAP- and DMSO-treated *PKAc-HA:loxP* schizonts undergoing egress. DMSO-treated schizonts are stained with Hoechst (blue). RAP, rapamycin.
(MP4)

S2 Movie Time-lapse video microscopy of RAP- and DMSO-treated *ACβ-HA:loxP* schizonts undergoing egress. DMSO-treated schizonts are stained with Hoechst (blue). RAP, rapamycin.
(MP4)

S3 Movie Time-lapse video microscopy of DMSO-treated *ACβ-HA:loxP* merozoites invading erythrocytes. (MP4)

S4 Movie Time-lapse video microscopy of RAP-treated *ACβ-HA:loxP* merozoites unable to invade erythrocytes following release from the schizont. RAP, rapamycin.
(MP4)

S5 Movie Time-lapse video microscopy of DMSO-treated *PKAc-HA:loxP* merozoites invading erythrocytes. (MP4)

S6 Movie Time-lapse video microscopy of RAP-treated *PKAc-HA:loxP* merozoites unable to invade erythrocytes following release from the schizont. RAP, rapamycin.
(MP4)

S7 Movie Electron tomography of the attachment region between a RAP-treated *PKAc-HA:loxP* merozoite and the RBC surface. Dual-axis tilt series were acquired from −60° to +60° with an increment of 2°. RAP, rapamycin; RBC, red blood cell.
(MP4)

## Abbreviations

ACα: adenylyl cyclase alpha
ACβ: adenylyl cyclase beta
AMA1: apical membrane antigen 1
ARO: armadillo repeats only protein
BiP: binding immunoglobulin protein
BsdR: blasticidin resistance selectable marker
cAMP: cyclic AMP
cGMP: cyclic GMP
Cas9: CRISPR-associated protein 9
CD: circular dichroism
CDPK: calcium-dependent protein kinase
DDD: DHFR destabilisation domain
DIC: differential interference contrast
DiCre: dimerisable Cre-recombinase
EBA175: erythrocyte binding antigen 175
EGFP: enhanced green fluorescent protein
EPAC: exchange protein directly activated by cAMP
E64: cysteine protease inhibitor
FV: food vacuole
GAPDH: glyceraldehyde 3-phosphate dehydrogenase
GAP45: glideosome-associated protein 45
glmS: glucosamine-6-phosphate riboswitch ribozyme
GST: glutathione S transferase
HA_3_: triple hemagglutinin
hDHFR: human dihydrofolate reductase selectable marker
HSQC: heteronuclear single quantum coherence
IFA: immunofluorescence assay
IMC: inner membrane complex
MyoA: myosin A
NMR: nuclear magnetic resonance
PDEβ: phosphodiesterase beta
PfEpac: *Plasmodium falciparum* EPAC
PI-PLC: phosphatidylinositol-specific phospholipase C
PKA: cAMP-dependent protein kinase
PKAc: catalytic subunit of cAMP-dependent protein kinase
PKAr: regulatory subunit of cAMP-dependent protein kinase
PKG: cGMP-dependent protein kinase
PPM: parasite plasma membrane
PV: parasitophorous vacuole
PVM: parasitophorous vacuole membrane
p50: processed 50 kDa form
RAMA: rhoptry-associated membrane antigen
RAP: rapamycin
RAP1: ras-related protein 1
RBC: red blood cell
sgRNA: single guide RNA
Rh2b: reticulocyte binding protein homologue 2b
ROM4: rhomboid protease 4
RON2: rhoptry neck protein 2
SERA2: serine repeat antigen 2
SERA5: serine repeat antigen 5
SLI: selection-linked integration
SMOAC: sequential metal oxide affinity chromatography
SUB1: subtilisin-like protease 1
SUB2: subtilisin-like protease 2
TEM: transmission electron microscopy
TMP: trimethoprim
T2A: thosea asigna virus 2A peptide
