# Supplementary figures and images for: Cyclic AMP signalling controls key components of malaria parasite host cell invasion machinery
Source: PLoS Biol. 2019 May 10;17(5):e3000264. doi: 10.1371/journal.pbio.3000264 (PMC6530879; doi:10.1371/journal.pbio.3000264)

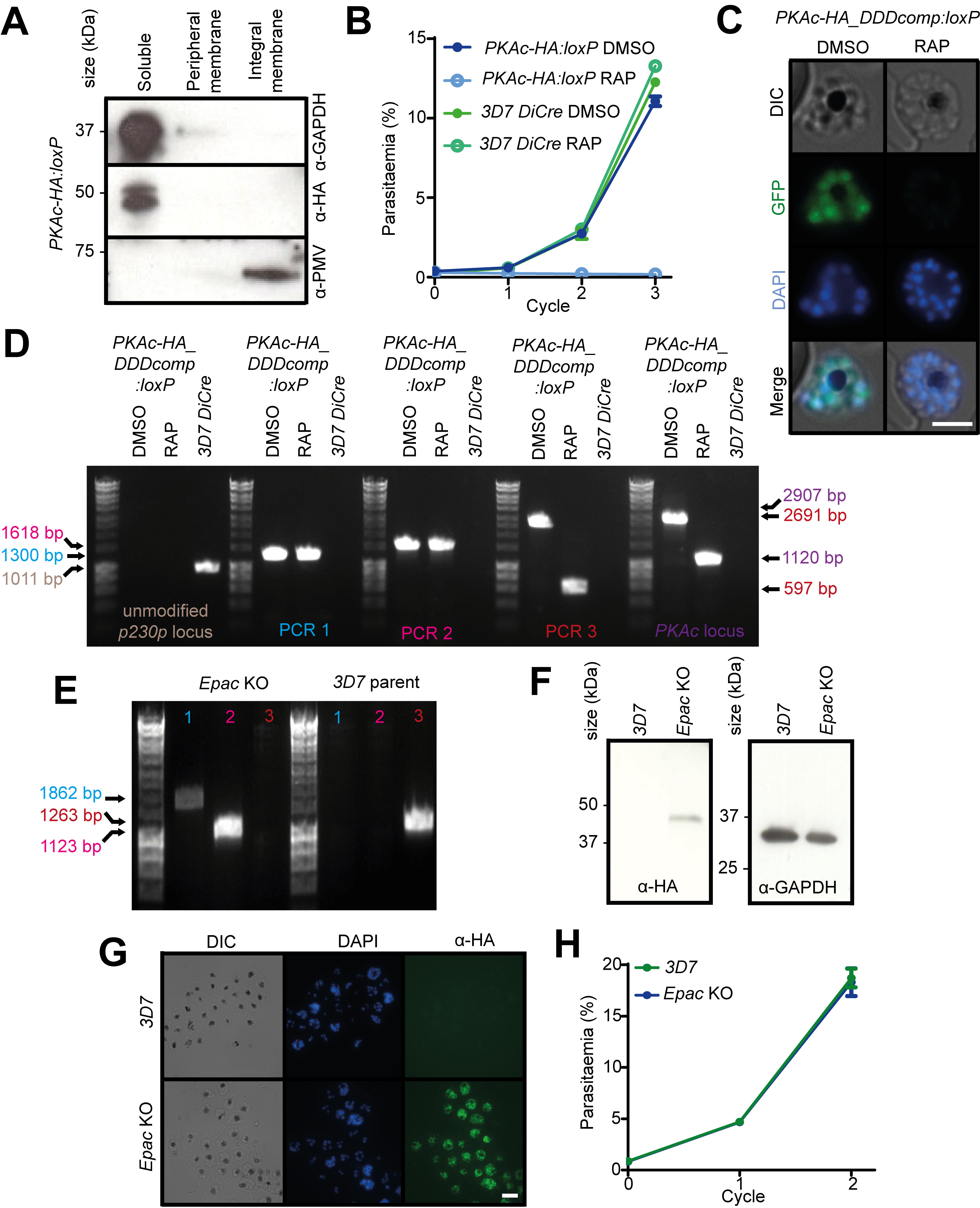

Supplement: S1 Fig — (A) Western blots from a subcellular fractionation experiment showing that PKAc-HA3 localises predominantly in the soluble fraction of a hypotonic freeze-thaw lysate of PKAc-HA:loxP schizonts. GAPDH was used as a positive control for the soluble fraction, and plasmepsin V (PMV) was used as a positive control for the integral membrane fraction, which was extracted with SDS/Triton X-144. The peripheral membrane fraction was extracted with 100 mM sodium bicarbonate. (B) Growth curves showing changes in parasitaemia of the parental 3D7 DiCre line and PKAc-HA:loxP parasites treated with DMSO (vehicle-only control) or RAP. Means from three replicates are plotted. Error bars, SD. (C) Fluorescence microscopy showing expression of EGFP in PKAc-HA_DDDcomp:loxP parasites, and subsequent loss of signal following RAP treatment, which switches expression of the protein(s) at this locus from EGFP expression to expression of PKAc-HA3_DDD. Scale bar, 50 μm. (D) Diagnostic PCR analysis verifying successful modification of the p230p locus of the PKAc-HA:loxP line to generate the PKAc-HA_DDDcomp:loxP line, and successful excision at the modified p230p and PKAc loci following treatment with RAP. Priming sites are indicated in Fig 3C, and the PCR used to amplify the PKAc locus corresponds to PCR 4 in Fig 2B. (E) Diagnostic PCR verifying successful integration of the transgene used to create the Epac knockout line. Priming sites are indicated in Fig 4D. (F) Western blot verifying expression of an approximately 42-kDa HA3-tagged fusion of the extreme N terminus of Epac upon deletion of the rest of the gene by the genetic modification shown in Fig 4D. GAPDH expression is shown as a loading control. (G) IFA verifying expression of an HA3 tag fused to the extreme N terminus of Epac upon deletion of the rest of the gene by the genetic modification shown in Fig 4D. Scale bar, 50 μm. (H) Growth curve showing unimpaired proliferation of PfEpac knockout parasites. Means from three replicates are p [file pbio.3000264.s001.tif]

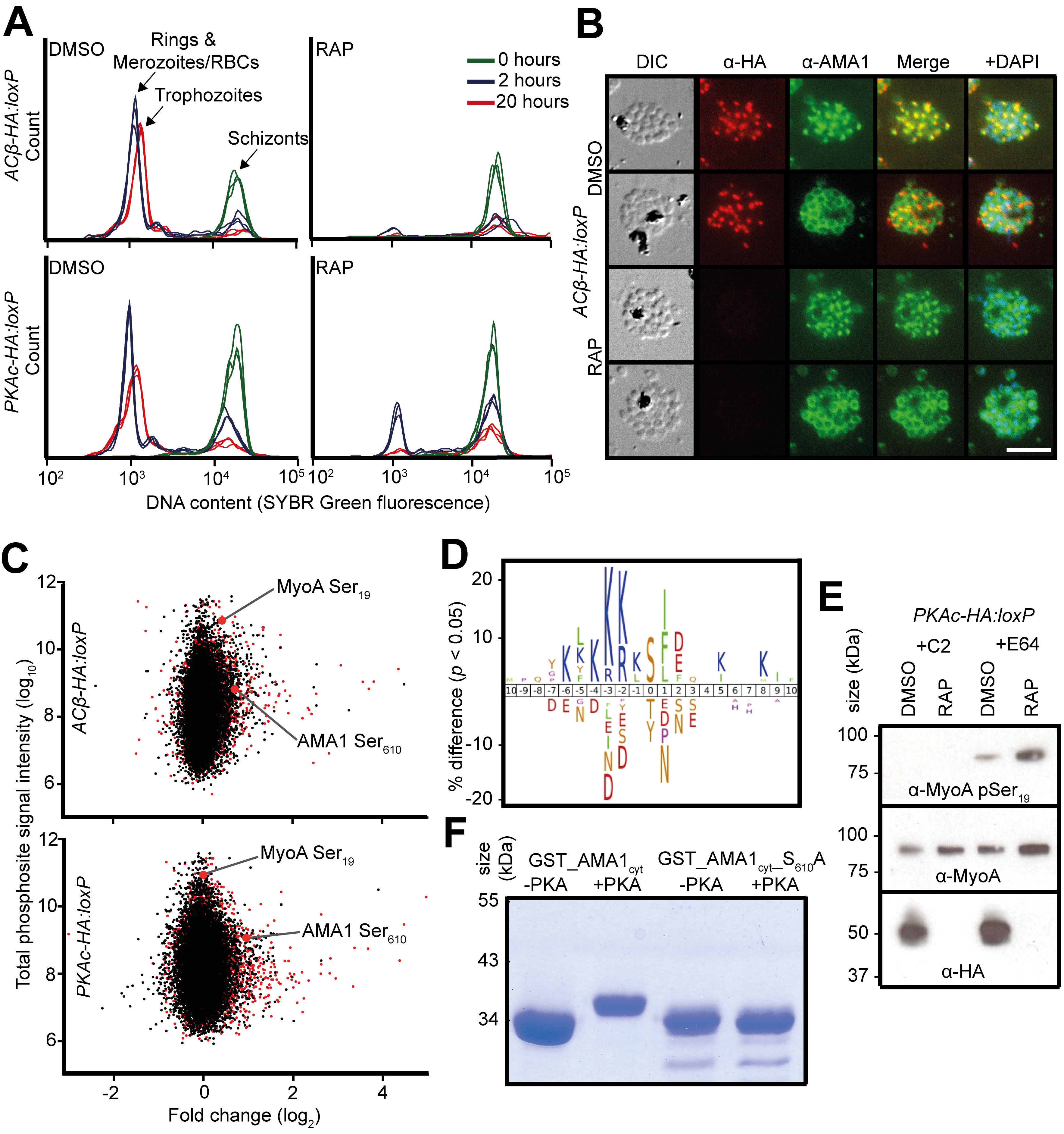

Supplement: S2 Fig — (A) Flow cytometry–based invasion assays showing the progression of DMSO-treated ACβ-HA:loxP and PKAc-HA:loxP parasites from schizonts (t = 0) through rings (t = 2) to trophozoites (t = 20), and the lack of progression of the RAP-treated counterparts through these stages. (B) IFA showing re-localisation of AMA1 from micronemes to the merozoite periphery in DMSO- and RAP-treated ACβ-HA:loxP schizonts. IFA analysis was performed on highly synchronous cultures, which were treated with 20 μM E64 about 44 h post invasion for approximately 4 h. Scale bars, 5 μm. (C) Ratio-intensity plots showing log10-transformed signal intensities plotted against the log2-transformed fold change in intensity (DMSO/RAP) for each site in the ACβ-HA:loxP and PKAc-HA:loxP phosphoproteomic profiling experiments. Sites that conform to a minimal PKA consensus motif (R/K, x, pS/pT) are indicated in red. (D) Motif analysis performed using IceLogo of the 533 31–amino acid regions surrounding phosphosites specifically enriched (Welch t test, p < 0.05) in DMSO-treated ACβ-HA:loxP− and PKAc-HA:loxP parasites compared with their RAP-treated counterparts. All 25,344 phosphosites detected in any sample were used as a reference dataset. Characters below the position line indicate amino acid residues that are unfavoured for those positions. (E) Western blot showing the presence of phosphorylated MyoA Ser19 in the absence of PKAc in the PKAc-HA:loxP line. (F) Coomassie stained gel showing changed mobility of GST-AMA1cyt following treatment with mouse PKA. This shift was not observed in the GST-AMA1cyt _S610A mutant. AMA1, apical membrane antigen 1; AMA1cyt, AMA1 cytosolic domain; E64, cysteine protease inhibitor; GST, glutathione S transferase; IFA, immunofluorescence assay; MyoA, myosin A; PKA, cAMP-dependent protein kinase; PKAc, catalytic subunit of cAMP-dependent protein kinase; RAP, rapamycin. (TIF) [file pbio.3000264.s002.tif]

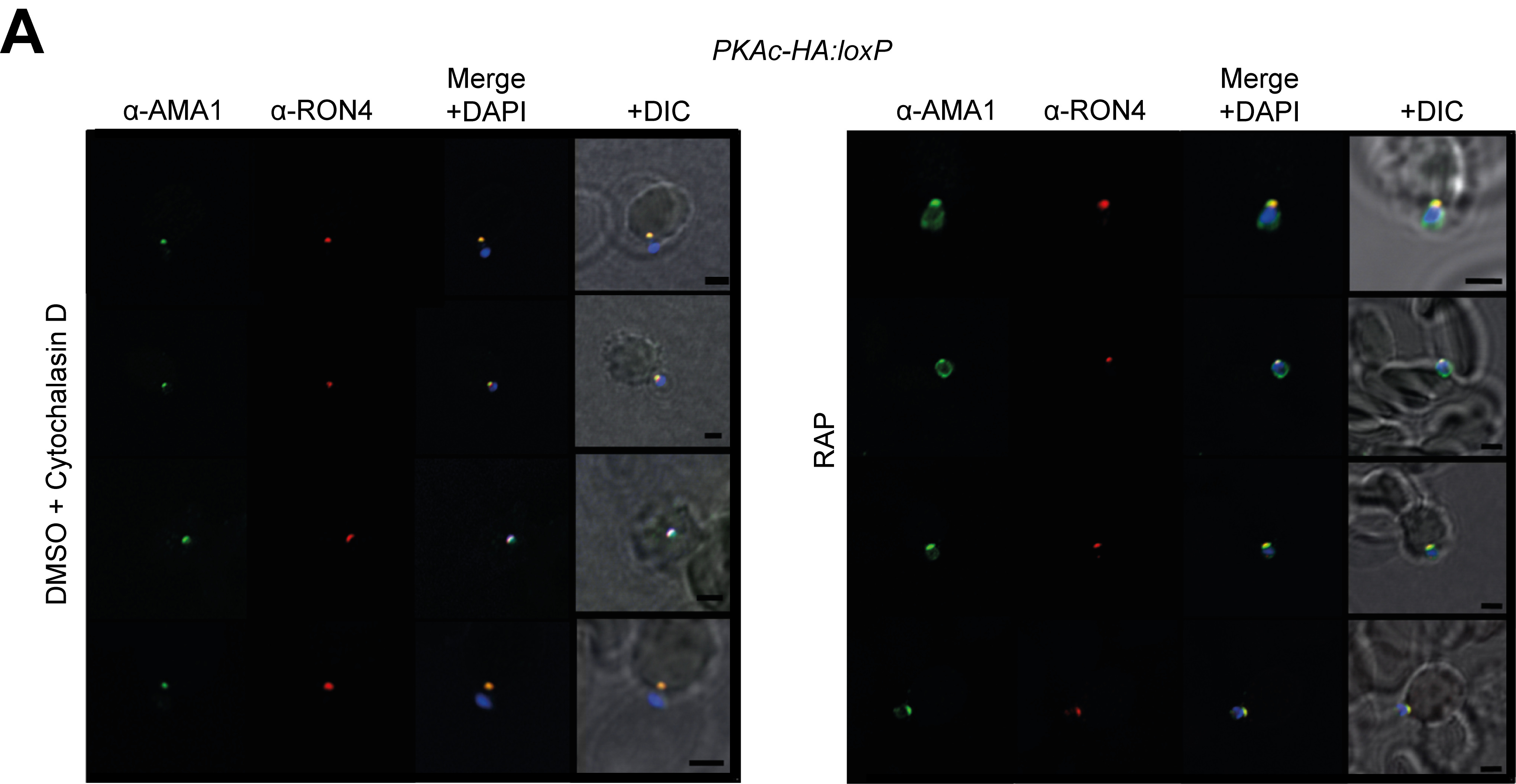

Supplement: S3 Fig — (A) Super-resolution immunofluorescence imaging of PKAc-HA:loxP merozoites attached to the RBC surface. Four merozoites for each condition (DMSO- or RAP-treated) are shown. Scale bars, 2 μm. RAP, rapamycin; RBC, red blood cell. (TIF) [file pbio.3000264.s003.tif]

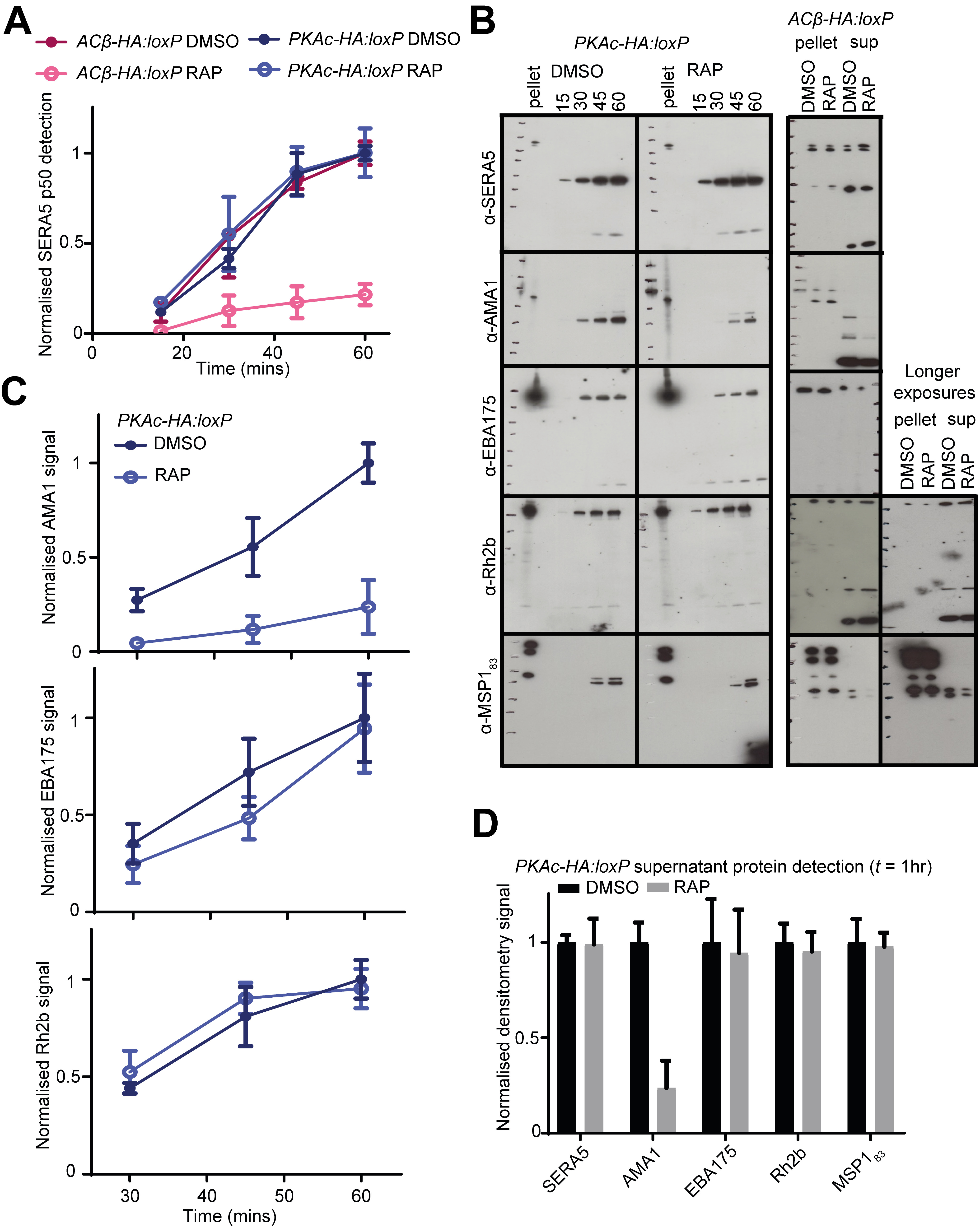

Supplement: S4 Fig — (A) Quantification of egress of DMSO- and RAP-treated PKAc-HA:loxP and ACβ-HA:loxP schizonts, based on densitometry measurements of the SERA5 p50 bands on the blots represented in Fig 4A. Signals are normalised such that the mean signal for the 60-min time point of each DMSO control is equal to one. Means from two replicates are plotted. Error bars, SD. (B) Full-length blots used to compile Fig 6A and Fig 6B. (C) Quantification of AMA1, EBA175, and Rh2b shedding from DMSO- and RAP-treated PKAc-HA:loxP merozoites, based on densitometry measurements on blots of the type represented in Fig 6A. Signals are normalised such that the mean signal for the 60-min time point of each DMSO control is equal to one. Means from three replicates are plotted. Error bars, SD. (D) Quantification of protein detection in the supernatants of rupturing DMSO- and RAP-treated PKAc-HA:loxP schizonts from blots of the type shown in Fig 6A. Densitometry measurements are normalised such that the mean signal for each DMSO control is equal to one. Means from three replicates are plotted. Error bars, SD. Data associated with this figure can be found in the supplemental data file (S1 Data). AMA1, apical membrane antigen 1; EBA175, erythrocyte binding antigen 175; p50, processed 50 kDa form; RAP, rapamycin; Rh2b, reticulocyte binding protein homologue 2b; SERA5, serine repeat antigen 5. (TIF) [file pbio.3000264.s004.tif]
